# Synthesis of graphene oxide-quaternary ammonium nanocomposite with synergistic antibacterial activity to promote infected wound healing

**DOI:** 10.1186/s41038-018-0115-2

**Published:** 2018-05-21

**Authors:** Tengfei Liu, Yuqing Liu, Menglong Liu, Ying Wang, Weifeng He, Gaoqiang Shi, Xiaohong Hu, Rixing Zhan, Gaoxing Luo, Malcolm Xing, Jun Wu

**Affiliations:** 1 Institute of Burn Research, State Key Laboratory of Trauma, Burn and Combined Injury, Southwest Hospital, Third Military Medical University (Army Medical University, Chongqing, China; 2 Chongqing Key Laboratory for Disease Proteomics, Chongqing, China; 3 Department of Mechanical Engineering, University of Manitoba, Winnipeg, MB Canada; 4 Department of Burns, The First Affiliated Hospital of Sun Yat-Sen University, Guangzhou, People’s Republic of China

**Keywords:** Graphene oxide, Quaternary ammonium salt, Antibacterial activity, Synergistic effect, Antibiotic resistance, Wound healing

## Abstract

**Background:**

Bacterial infection is one of the most common complications in burn, trauma, and chronic refractory wounds and is an impediment to healing. The frequent occurrence of antimicrobial-resistant bacteria due to irrational application of antibiotics increases treatment cost and mortality. Graphene oxide (GO) has been generally reported to possess high antimicrobial activity against a wide range of bacteria *in vitro*. In this study, a graphene oxide-quaternary ammonium salt (GO-QAS) nanocomposite was synthesized and thoroughly investigated for synergistic antibacterial activity, underlying antibacterial mechanisms and biocompatibility *in vitro* and *in vivo*.

**Methods:**

The GO-QAS nanocomposite was synthesized through amidation reactions of carboxylic group end-capped QAS polymers with primary amine-decorated GO to achieve high QAS loading ratios on nanosheets. Next, we investigated the antibacterial activity and biocompatibility of GO-QAS *in vitro* and *in vivo*.

**Results:**

GO-QAS exhibited synergistic antibacterial activity against bacteria through not only mechanical membrane perturbation, including wrapping, bacterial membrane insertion, and bacterial membrane perforation, but also oxidative stress induction. In addition, it was found that GO-QAS could eradicate multidrug-resistant bacteria more effectively than conventional antibiotics. The *in vitro* and *in vivo* toxicity tests indicated that GO-QAS did not exhibit obvious toxicity towards mammalian cells or organs at low concentrations. Notably, GO-QAS topically applied on infected wounds maintained highly efficient antibacterial activity and promoted infected wound healing *in vivo*.

**Conclusions:**

The GO-QAS nanocomposite exhibits excellent synergistic antibacterial activity and good biocompatibility both *in vitro* and *in vivo*. The antibacterial mechanisms involve both mechanical membrane perturbation and oxidative stress induction. In addition, GO-QAS accelerated the healing process of infected wounds by promoting re-epithelialization and granulation tissue formation. Overall, the results indicated that the GO-QAS nanocomposite could be applied as a promising antimicrobial agent for infected wound management and antibacterial wound dressing synthesis.

**Electronic supplementary material:**

The online version of this article (10.1186/s41038-018-0115-2) contains supplementary material, which is available to authorized users.

## Background

Antibiotics have saved countless lives since their first appearance. However, the prevalence of antimicrobial-resistant bacteria, caused by the indiscriminate application and overuse of antibiotics, has been a global issue threatening human health for the past several decades [1]. Antimicrobial resistance is projected to cause the death of approximately 300 million people in the next 35 years [2]. Wound infection with antimicrobial-resistant bacteria aggravates wound deterioration, delays wound healing, and even causes sepsis and death [3]. Thus, it is imperative for us to find alternative ways to prevent and treat wound infection to restore the normal microenvironment for wound healing.

In recent years, graphene oxide (GO) nanosheets have drawn much attention due to their excellent antibacterial activity [4–6]. Several reports have claimed that GO-based nanocomposites, whether topically applied on wounds as antibacterial agents [7] or incorporated into dressing scaffolds as antibacterial wound dressings [8, 9], can significantly facilitate wound healing by eradicating bacteria in wounds. GO, a derivative of graphene, is a two-dimensional one-atom-thick sheet composed of sp^2^-hybridized carbon atoms [10]. The observed antimicrobial properties of GO have been attributed to the combined mechanisms of bacterial membrane perturbation caused by sharp edges and oxidative stress induction [11, 12]. Additionally, an abundance of functionalized hydroxyl, epoxy, and carboxyl groups on the graphene oxide surface enhance its hydrophilicity and biocompatibility and significantly facilitate its surface modification with other molecules or polymers [4, 5, 10]. However, owing to the presence of strong interplanar interactions, graphene oxide is prone to aggregate in aqueous solutions in a layer by layer manner, which potentially limits its application [13, 14]. Furthermore, there is currently no consensus regarding the intrinsic antibacterial properties of bare graphene oxide [15, 16]. Different reports claim that GO possesses strong [4, 11], very weak [17], or no [18] antimicrobial activity, or even facilitates bacterial proliferation [19]. Therefore, it is necessary to prepare functionalized graphene oxide derivatives to consolidate the antibacterial activity of graphene oxide and make it more stable in aqueous solutions.

Currently, there are several reports on avoiding graphene oxide aggregation by grafting certain materials, such as polydopamine [20], Fe_3_O_4_ [21], and silver nanoparticles (AgNPs) [22, 23]. However, the cytotoxicity of AgNPs is a matter of concern [24]. Quaternary ammonium salts (QAS) are other commonly utilized bactericides that exhibit strong antibacterial activity [25–27]. The general antibacterial mechanism of QAS is believed to be associated with adsorption on the cell wall through electrostatic interactions, which subsequently increases membrane permeability and disrupts the membrane [25–27]. Two-dimensional GO nanosheets have an intrinsically high surface area to volume ratio and therefore could be an ideal nanocarrier platform to load QAS at a high density, allowing a larger contact interface with bacteria to advance the antibacterial performance [28]. Moreover, QAS can also be used as cationic surfactants to facilitate dispersion of GO nanocarriers [27]. It is worthwhile to explore the enhanced antimicrobial effects of graphene oxide-quaternary ammonium salts (GO-QAS) nanocomposites. Recently, Ye et al. reported the synthesis of graphene oxide/reduced graphene oxide-QAS nanocomposites with significant antibacterial activity through π − π conjugations [29, 30]. Tu et al. reported a click synthesis of a GO-quaternized poly(dimethylaminoethyl methacrylate) nanocomposite coating with good antibacterial and antifouling ability [31]. Despite these reports concerning the synthesis of GO-QAS with good antibacterial activity, it is still unclear whether GO-QAS has an advantage as a new antibacterial nanomaterial weapon with unique or synergistic efficiency compared with the simple combination of separate GO and QAS, and the detailed antibacterial mechanisms of GO-QAS nanocomposites are still unknown. In addition, little is known about the biocompatibility and antibacterial activity of GO-QAS *in vivo*.

To address these questions, in this work, GO-QAS nanocomposites were synthesized through amidation reactions of carboxylic group end-capped QAS polymers with primary amine-decorated GO to achieve high QAS loading ratios on nanosheets, and we investigated, for the first time, the synergistic antibacterial activity of GO-QAS and the underlying antibacterial mechanisms. We demonstrated that compared with pure GO, QAS or the simple mixture of GO and QAS, GO-QAS with an optimal QAS graft ratio exhibited significantly enhanced and synergistic antibacterial activity. The antibacterial mechanisms of GO-QAS involved not only mechanical bacterial membrane perturbation, including wrapping, bacterial membrane insertion, and bacterial membrane perforation, but also oxidative stress induction. In addition, GO-QAS exhibited significant antibacterial activity against Methicillin-resistant *Staphylococcus aureus* (MRSA) and multidrug-resistant *Acinetobacter baumanii* (MDR-AB). Furthermore, we demonstrated the good biocompatibility of GO-QAS both *in vitro* and *in vivo*. Moreover, when topically applied on infected wounds *in vivo* using a murine-infected full-thickness skin defect wound model, GO-QAS could eradicate pathogenic bacteria on wounds and facilitate the healing of infected wounds. To the best of our knowledge, this study represents the first systematic investigation of the antimicrobial and biocompatibility properties of GO-QAS both *in vitro* and *in vivo*. We expect our research to provide an in-depth understanding of the antibacterial behaviors and biocompatibility of GO-QAS and to serve as a practical reference for GO-QAS-based antibacterial wound dressings or antifouling coating materials research.

## Methods

### Materials

GO powder (1-5 layers, 95 wt%, specific area 300–450 m^2^/g) was purchased from Suzhou Tanfeng Ltd. (Suzhou, China). Double deionized water (DD water) was purified with a Millipore Q3 instrument. LIVE/DEAD™ BacLight™ Bacterial Viability kit (L7012, Molecular Probes, Invitrogen) was purchased from Thermo Fisher Scientific (Life Technologies, USA). All other chemicals were purchased from Sigma-Aldrich and used directly.

BALB/c mice (male, 20–25 g) were purchased from the Experimental Animal Department of the Army Medical University (Chongqing, China) and were raised individually in plastic cages under standardized conditions (room temperature: 25 °C; relative humidity: 50%; and circadian rhythm: 12 h) for 2 weeks before the experiment, with free access to autoclaved standard rodent chow and water. All the animal experiments (including *in vivo* biosafety, antibacterial activity, and infected wound healing evaluation) were approved by the Institutional Animal Care and Use Committee of the Third Military Medical University.

### Synthesis of graphene oxide-quaternary ammonium salts nanocomposite

#### Synthesis of S-dodecyl-S′-(α,α′-dimethyl-α″-acetic acid) trithiocarbonate (RAFT-COOH)

Synthesis of the RAFT-COOH chain transfer agent was in accordance with previously published literature [32].

#### Synthesis of (2-(acryloyloxy)ethyl)-N,N-dimethylhexa-ammonium bromide (AEDMHA)

The quaternary ammonium salt monomers were prepared by quaternization of 1-bromohexane with 2-(dimethylamino)ethyl acrylate (DMAEA). Briefly, 1.43 g DMAEA (10 mmol) and 1.98 g 1-bromohexane (12 mmol) were added into a 25 mL round-bottom flask filled with 10 mL of acetonitrile (MeCN), and the solution was heated at 80 °C for 16 h. The reaction was quenched by soaking in an ice water bath. Most of the solvent was removed by rotational evaporation, and 20 mL of ethyl ether was added into the flask to wash the mixture. AEDMHA was crystallized at 4 °C, filtered and rinsed with cold ethyl ether. The pale powder was recovered by drying in a vacuum oven overnight, with a yield of ~ 2.6 g.

#### RAFT polymerization of AEDMHA quaternary ammonium salt (PAEDMHA)

Briefly, 1.5 g AEDMHA monomer, 68.3 mg RAFT-COOH, 6.2 mg azobisisobutyronitrile (AIBN), and 5 mL of MeCN were added into a 10 mL round-bottom flask sealed with a rubber septum to target an average molecular weight of 8 kDa. The solution was degassed by nitrogen sparging for 20 min and stirred at 60 °C for 20 h. After the reaction, a small portion of the solution was collected, dried under vacuum, and used for measuring polymerization conversion via ^1^H nuclear magnetic resonance (NMR) spectroscopy. The product was precipitated out of tetrahydrofuran (THF) twice for purification and recovered from drying under vacuum as a light-yellow powder with a yield of 1.05 g.

#### Amine-grafted graphene oxide (GO-NH_2_) via silanization

The salinization of GO was conducted in dimethylformamide (DMF). Briefly, 150 mg of GO powder was mixed with 40 mL DMF and 1.2 mL DD water. The mixture was dispersed using a probe sonic homogenizer (SK92-IIN ultrasonicator, maximum output power 650 W, 20–25 kHz) with an output of 50%, in an ice bath for 2 h. After sonication, there were no visible particles of GO in the mixture. Then, 0.6 mL of (3-aminopropyl)trimethoxysilane (APTMS) was added into the mixture under nitrogen protection. The dispersion was stirred at 60 °C for 15 h, and the dispersed mixture became very viscous after the reaction. GO-NH_2_ was precipitated out of ethanol, collected via centrifugation, and repeatedly washed with ethanol. The product was dispersed in water and dialyzed against DD water with dialysis tubing (molecular weight cutoff (MWCO) = 12~14 kDa) for 3 days, followed by lyophilization. Dark gray powder was collected with a yield of ~ 350 mg.

#### Preparation of quaternary ammonium salt polymer-modified GO

PAEDMHA polymers were grafted onto GO-NH_2_ nanosheets via an amidation reaction with different grafting ratios, and the feeding ratios (mass) of GO-NH_2_:PAEDMHA were 1:2, 1:4, 1:8, and 1:16, respectively, for GO-QAS-1, GO-QAS-2, GO-QAS-3, and GO-QAS-4. In the typical reaction of GO-QAS-3, 100 mg of GO-NH_2_ powder, 800 mg of PAEDMHA polymer, and 40 mL of DD water were mixed together and sonicated using a probe sonic homogenizer for 3 h under the same conditions described above. To the dispersed mixture, 33.2 mg of 1-ethyl-3-(3-dimethylaminopropyl)carbodiimide hydrochloride (EDC) and 20.2 mg of N-hydroxysuccinimide (NHS) were added. Then, the dispersion was stirred at ambient temperature for 2 days. The mixture was further sonicated for 30 min after the first 24-h reaction. After the reaction, the dispersion was dialyzed against DD water with dialysis tubing (MWCO = 12~14 kDa) for 3 days to remove unconjugated polymers, and the final product GO-poly (AEDMHA) was recovered by lyophilization with a yield of 535 mg as a dark gray powder. For other reactions, the ratio of GO-NH_2_:PAEDMHA changed, but the ratio of PAEDMHA:EDC/NHS was kept the same. The product yields and estimated grafted ratios are presented in Table S1 (see Additional file 1). For convenience, GO-QAS was used to denote GO- poly (AEDMHA) in our article.

### Characterization

Nuclear magnetic resonance (NMR). All ^1^H NMR tests were carried out on a Bruker Avance 300 MHz NMR spectrometer using CDCl_3_ or D_2_O as the solvent at a concentration of 10 mg/ml, with 32 scans and a relaxation delay (d1) set to 1 s.

Fourier transform infrared (FTIR) spectroscopy*.* FTIR-ATR (attenuated total reflection) characterizations were conducted on a Nicolet iS10 FTIR spectrometer, with 32 scans and a resolution of 4.

Atomic force microscopy (AFM)*.* The thickness of GO and GO-QAS nanosheets was characterized by AFM. Briefly, a single drop of dispersion was spread on the surface of freshly cut mica and air-dried prior to AFM analysis.

Scanning electron microscopy (SEM). Briefly, single drops of GO and GO-QAS dispersions were drop–cast onto a clean silicon wafer and air-dried. Then, samples were Au sputter-coated for 60 s before SEM imaging (Zeiss Crossbeam 340, Germany).

Transmission electron microscopy (TEM)*.* The morphology of GO and GO-QAS nanosheets was characterized by TEM. Briefly, a drop of the prepared dispersion was deposited on a carbon-coated copper grid and air-dried preceding TEM (JEOL JEM-1400, Japan) analysis.

Dynamic light scattering (DLS). The particle size distribution of GO and GO-QAS was determined with a particle size analyzer (Malvern Zetasizer Nano ZS90, Britain). A refractive index (*n* = 1.3) matching the filtered (0.2 μm) bath surrounded the scattering cell, and the temperature was fixed at 25 °C. Three independent measurements were performed for each sample.

Zeta potential measurements. Zeta potential measurements of GO and GO-QAS dispersions at 50 μg/mL in deionized (D.I.) water were performed on a zeta-potential analyzer (Malvern Zetasizer Nano ZS90, Britain) at 25 °C. Each measurement was repeated at least three times.

Thermogravimetric analysis (TGA). TGA was conducted using a TGA Q500 thermogravimetric analyzer from room temperature to 600 °C at a heating rate of 10 °C/min under a nitrogen atmosphere. All samples were completely dried under vacuum without heating and stored in a desiccator before the test.

### Antimicrobial activity investigation

#### Bacterial cells and nanocomposite preparations

Four representative bacterial species, *Staphylococcus aureus* (*S. aureus*, ATCC 25923), *Escherichia coli* (*E. coli*, ATCC 25922), MRSA (ATCC 43300), and a clinically isolated MDR-AB, were chosen as model gram-positive and gram-negative bacteria in the present study. Briefly, a single isolated colony of bacteria on a solid Luria-Bertani (LB) agar plate was transferred to 5 mL of LB broth medium and grown overnight at 37 °C under 200 rpm rotation. Then, the bacteria culture medium was centrifuged at 6000 rpm for 5 min, and the bacterial pellet was subsequently washed three times with deionized water to remove medium constituents and other chemical macromolecules [33]. Afterwards, the pellet was resuspended in deionized water, and the optical density (OD) value of the suspension was adjusted to 0.5 at 600 nm, which corresponded to a concentration of 10^8^ colony-forming units per milliliter (CFU/mL). The stock dispersions of all the nanocomposites were prepared at a concentration of 1 mg/mL and then diluted to certain concentrations. All the nanocomposite dispersions were sterilized by UV radiation for 30 min preceding experiments.

#### Influence of the QAS graft ratio on the antibacterial activity of GO-QAS

To determine the optimal QAS graft ratio at which the GO-QAS nanocomposite exhibited the strongest antibacterial activity, we investigated the antibacterial activity of GO-QAS with diverse QAS graft ratios using the plate count method as previously reported [34]. Briefly, 200 μL aliquots of *E. coli* and *S. aureus* bacteria suspensions at 0.5 OD600_nm_ in deionized water were mixed with 20 μL of GO-QAS dispersions with diverse QAS graft ratios at 50 μg/mL concentrations (GO-QAS-1; GO-QAS-2; GO-QAS-3; GO-QAS-4) and then incubated at 37 °C for 3 h with constant shaking at 200 rpm. Control samples were prepared with 200 μL cell suspensions and 20 μL deionized water. After 3 h, serial 10-fold dilutions of cells were spread onto LB plates and left to grow overnight at 37 °C. Colonies were counted, and the numbers were compared with control plates to calculate the cell viability rate. All treatments were prepared in triplicate and repeated on three separate occasions. The cell viability rate was calculated based on the following formula: cell viability % = counts of test samples/counts of control×100%.

#### Antibacterial activity of the optimized GO-QAS nanocomposite at different concentrations

##### Agar diffusion test

The inhibition zone of GO-QAS at different concentrations was determined by an agar diffusion test as previously reported, with some modifications [35]. Briefly, 100 μL of bacterial suspensions (10^8^ CFU/mL) of *S. aureus* and *E. coli* were uniformly spread on the surface of Muller Hinton agar plates with sterile cotton swabs. Then, six wells (diameter of 6 mm) were made on each test plate with the help of a sterile borer. Samples (50 μL) of GO and GO-QAS dispersions at different concentrations (from 5 to 200 μg/mL) were introduced into the peripheral five wells. An equal volume of streptomycin sulfate (1 mg/mL) was loaded in the central well to serve as the positive control. Then, all the plates were incubated at 37 °C overnight. The antimicrobial activity was observed as transparent halos surrounding the well.

##### Plate count method

The plate count method was performed to determine the antibacterial activity of GO, QAS, and GO-QAS dispersions at different concentrations as mentioned above. Briefly, 200 μL of cell suspension was mixed with 20 μL of different concentrations of GO, QAS, and GO-QAS dispersions (i.e., 50, 100, and 200 μg/mL) and then incubated for 3 h at 37 °C under 200 rpm rotation. Deionized water was used as the control. After 3 h, diluted cells were spread onto LB plates and left to grow overnight at 37 °C. Colonies were counted, and the numbers were compared with control plates to calculate the cell viability rate.

##### Bacterial live/dead viability assay

The antibacterial activity of GO, QAS, and GO-QAS was further verified with a bacterial live/dead viability assay [36]. The dyes SYTO 9 and propidium iodide (PI) were used according to the manufacturer’s instructions. Briefly, 200 μL of cell suspension was mixed with 20 μL of GO, QAS, or GO-QAS dispersions at 200 μg/mL concentration (selected based on the highest antimicrobial activity) or control (sterile deionized water). The mixture was incubated at 37 °C for 3 h at 200 rpm. Then, cells were harvested by centrifugation in a microcentrifuge tube at 6000 rpm and resuspended in 1 mL of sterile deionized water. Then, the suspensions were stained with SYTO 9 (3 μL) and PI (3 μL) for 15 min in the dark. Afterwards, a 2 μL aliquot of stained suspension was dropped on a clean glass slide and imaged under a laser confocal microscope (Zeiss LSM780, Germany). The cell death percentage is expressed as the number of dead cells (in red)**/**the number of total cells (in green)×100%.

#### Investigation of the synergistic antibacterial effect of GO-QAS

The synergistic antibacterial effect of GO-QAS was investigated using the plate count method as previously reported [37]. *S. aureus* and *E. coli* bacteria cells were treated with dispersions of 8 μg/mL GO, 81.3 μg/mL QAS, a mixture of 8 μg/mL GO and 81.3 μg/mLQAS, or 100 μg/mL GO-QAS. After incubation, diluted cells were spread onto LB plates and left to grow overnight at 37 °C. Colonies were counted, and the numbers were compared with control plates to calculate the cell viability rate.

#### Antimicrobial property of GO-QAS against multidrug-resistant bacteria

The antimicrobial property of GO-QAS against MRSA and MDR-AB was determined by the two-fold dilution method [38]. Bacteria (OD600_nm_ = 0.5) were exposed to a two-fold serial dilution of GO-QAS (from 0 to 200 μg/mL) in LB broth and incubated at 37 °C for 24 h. Penicillin and streptomycin were used as the positive control for MRAS and MDR-AB, respectively. After 24 h of incubation, the optical density at 600 nm was measured with an enzyme-linked immunosorbent assay microplate reader, and bacterial cells were spread onto agar plates after serial dilution for colony counting.

### Investigation of the antibacterial mechanisms of GO-QAS

#### Cell morphology observation with SEM

The morphological changes in bacterial cells after GO, QAS, and GO-QAS treatments were observed with SEM as previously reported [4]. *E. coli* was chosen as the bacteria for the experiment. Briefly, after incubation with GO, QAS, and GO-QAS dispersions at 200 μg/mL or control (sterile deionized water) for 2 h, 100 μL of the mixture was diluted to a 1 mL volume. Then, 20 μL of the bacteria-nanocomposite mixture was dropped on the surface of sterile glass coverslips, fixed with 2.5% glutaraldehyde for 2 h at room temperature, and washed twice with 0.9% NaCl solution, followed by dehydration in a graded ethanol series and drying overnight at room temperature. The glass coverslips were then Au sputter-coated for 60 s before imaging via SEM (Zeiss Crossbeam 340, Germany) under high-vacuum conditions at an accelerating voltage of 2.0 kV.

#### Reactive oxygen species (ROS) detection

The oxidation-sensitive fluorescent dye 2^′^, 7^′^-dichlorofluorescin diacetate (DCFH-DA, Sigma-Aldrich, USA) was utilized to detect reactive oxygen species generation in the bacteria suspension according to a previously reported protocol [35]. In brief, 2 mL of *E. coli* bacterial suspensions were incubated with 200 μL of GO, QAS, and GO-QAS dispersions at 200 μg/mL or the control (deionized water) for 2 h. Then, the cells were washed three times with 0.1 M PBS (pH 7.8) and resuspended in 2 mL of PBS solution. Then, 2 μL of DCFH-DA (10 mM) was added to make the final concentration of DCFH-DA 10 μM in the mixture. Afterwards, the mixture was incubated in the dark for 1 h, centrifuged, and washed to remove the unloaded DCFH-DA probe. The fluorescence intensity was measured on an enzyme-linked immunosorbent assay microplate reader (Thermo Varioskan Flash, USA) at an excitation wavelength of 488 nm and emission wavelength of 525 nm. The intracellular ROS level is expressed as the percentage of the fluorescence intensity of the samples to that of the control.

#### Measurement of cytoplasmic DNA and RNA efflux

Bacterial cell membrane integrity was examined by detecting the release of cytoplasmic nucleic acids (DNA and RNA) at 260 nm absorption according to a previous protocol [39]. Briefly, 2 mL of *E. coli* bacterial suspension was incubated with 200 μL of GO, QAS, and GO-QAS dispersions at 200 μg/mL or the control (deionized water) for 2 h at 37 °C. The mixture was then immediately filtered with 0.2-μm syringe filters to remove bacteria and nanocomposites. The efflux of cytoplasmic nucleic acids from the bacteria into the supernatant was measured with an ND-1000 spectrophotometer (NanoDrop) at 260 nm.

### *In vitro* cytotoxicity and hemolysis assay

#### Cell culture

A human immortalized keratinocyte (HaCaT) cell line was utilized for cytotoxicity investigation of GO and GO-QAS. Cells were cultured in Dulbecco’s modified Eagle’s medium (DMEM) supplemented with 10% fetal bovine serum (FBS), penicillin (100 U/mL), and streptomycin (100 μg/mL) in a 5% CO_2_ incubator at 37 °C.

#### Cell viability test

Cell viability was detected with a Cell Counting Kit-8 (CCK-8) assay based on a previously reported method [35]. In brief, 3 × 10^4^ cells in 150 μL of DMEM supplemented with 10% FBS were seeded into 96-well plates and incubated in a 5% CO_2_ incubator at 37 °C for 24 h. Then, the culture medium was discarded, and HaCaT cells in the plates were gently rinsed twice with sterile PBS, followed by addition of GO and GO-QAS dispersions at different concentrations or control (deionized water) to the medium and another 24 h of incubation at 37 °C. After incubation, the culture medium was aspirated, and cells were washed twice with sterile PBS, followed by addition of 10 μLL of CCK-8 reagent (Dojindo, Kyushu, Japan) and 100 μL of fresh DMEM. After 4 h of further incubation at 37 °C, the sample absorbance was quantitated at 450 nm using an enzyme-linked immunosorbent assay microplate reader (Thermo Varioskan Flash, USA).

#### Flow cytometry apoptosis assay

To further investigate the cytotoxicity of GO-QAS, an Annexin V-FITC apoptosis detection kit (C1062, Beyotime, China) was used to detect the ratio of apoptotic and necrotic cells as previously reported [40, 41]. Briefly, after incubation with GO, GO-QAS, or control (deionized water) for 24 h, HaCaT cells were collected, washed with cold PBS solution, and resuspended in 195 μL of binding buffer, followed by successive addition of 5 μL of Annexin V-FITC and 10 μL of PI. Then, the mixture was incubated for 15 min at room temperature in the dark and analyzed with a fluorescence-activated cell sorting (FACS) sorter (Attune acoustic focusing cytometer). The Annexin V-FITC^+^ and PI^−^ quadrant represented the apoptotic cells, while the Annexin V-FITC^+^ and PI^+^ quadrant represented the necrotic cells.

#### *In vitro* hemolysis assay

The hemolysis assay was carried out based on a previously reported method [42]. A fresh human blood sample was collected from a healthy volunteer in the Southwest Hospital with the donor’s consent. Erythrocytes were collected by centrifugation at 1500 rpm for 15 min and washed three times with saline. Then, 1 mL of the centrifuged erythrocytes was mixed with 3.67 mL of saline. Afterwards, 100 μL of diluted erythrocyte dispersion was added to 1 mL of GO and GO-QAS dispersions in saline at different concentrations. Saline and deionized water served as the negative and positive control. The mixed dispersions were incubated at 37 °C for 3 h. The hemolysis ratio was evaluated by measuring the supernatant at 540 nm absorbance with an enzyme-linked immunosorbent assay microplate reader (Thermo Varioskan Flash, USA) after centrifugation at 12000 rpm for 15 min. The hemolysis ratio was calculated based on the following formula:

$$ \mathrm{Hemolysis}\ \mathrm{ratio}\%=\left({A}_S-{A}_N\right)/\left({A}_P-{A}_N\right)\times 100\% $$

where *A*_*S*_ represented the absorbance of the corresponding nanocomposite, *A*_*N*_ was the absorbance of the negative control, and *A*_*P*_ represented the absorbance of the positive control.

### *In vivo* biosafety evaluation

To investigate the biocompatibility of GO-QAS *in vivo*, mice were intravenously administered 20 μL of sterile PBS buffer (10 mM, pH 7.4) or GO and GO-QAS dispersions at 100 μg/mL through the tail vein [43]. Seven days post administration, the mice were sacrificed and organs (including the liver, kidney, spleen, and heart) were harvested. Then, the organs were fixed with 4% paraformaldehyde, embedded in paraffin, sectioned at a thickness of 5 μm, and stained with hematoxylin and eosin (H&E).

### *In vivo* effect of GO-QAS on wound infection

#### *In vivo* antimicrobial evaluation and wound healing

To evaluate the antimicrobial effect of GO-QAS *in vivo*, a murine-infected full-thickness skin defect wound model was constructed [44]. Briefly, BALB/c mice were intraperitoneally anesthetized with 1% pentobarbital. The dorsal fur was shaved, and the skin was cleaned with 75% alcohol. On each side of the back, one 6-mm-diameter full-thickness wound was created with a punch. A 6-mm-diameter sterile round marker was placed beside each wound to represent the initial wound area and the wounds were photographed immediately using a digital camera. Twenty mice were enrolled in the study and randomly divided into blank, control, GO and GO-QAS groups, with five mice in each group. GO, GO-QAS and control groups were inoculated with a 5 μL aliquot of an *S. aureus* and *E. coli* mixed suspension (at 0.5 McFarland standard), and each wound was treated with 5 μL of GO or GO-QAS dispersions at 100 μg/mL (selected based on the cytotoxicity results) or deionized water (control group), respectively. Blank group without bacterial inoculation was used as the negative control. Each wound was then covered with a sterile gauze. Afterwards, a piece of biological membrane (NPWT-1, Negative Pressure Wound Therapy Kit, China) was pasted onto the surface of the gauze. Mouse wounds without infection were used as the negative control. At days 3, 5, and 7 post-infection, the wounds were photographed, and the number of bacteria on the wound was quantified using the plate count method. Wound areas were measured using IPP 6.0 software. The wound healing rate was calculated based on the following formula:

$$ \mathrm{Wound}\ \mathrm{healing}\ \mathrm{rate}\%=\left(I-R\right)/I\times 100\% $$

where *I* represented the initial wound area and *R* represented the wound area that remained on the determined day post-infection [45].

#### Histological analysis of wound tissues

For histological analysis, mice were sacrificed on day 3 or 7 post-surgery. The skin wound specimens were harvested, fixed with 4% paraformaldehyde, embedded in paraffin, cut at a thickness of 5 μm, and then stained with H&E. The length of the neo-epithelium and the granulation tissue thickness were measured using ImageJ software. The length of the neo-epithelium is defined as the distance between the wound margin and the advancing edge of the epidermis [45]. All software measurements were performed by two independent researchers.

## Results

### Synthesis and characterization of antibacterial GO-QAS nanosheets

**Scheme 1:**
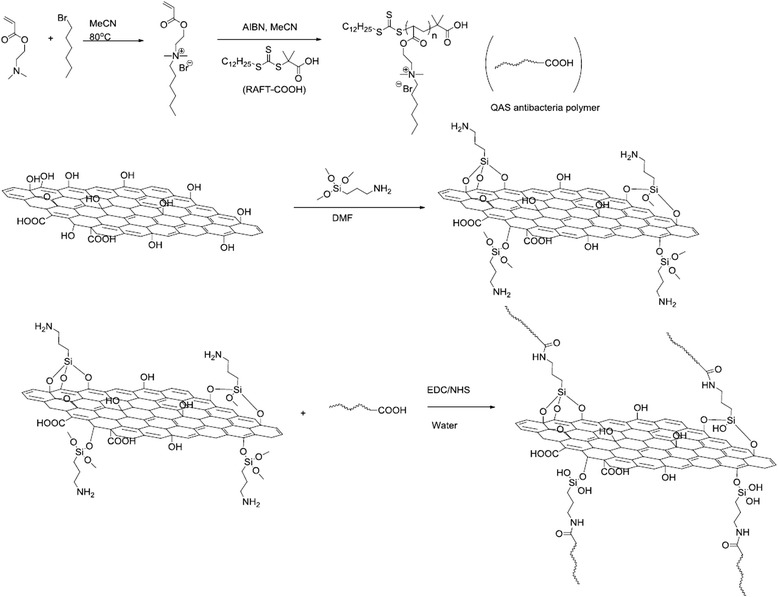
Synthetic route of graphene oxide-quaternary ammonium salt (GO-QAS) antibacterial nanocomposite

**Fig. 1:**
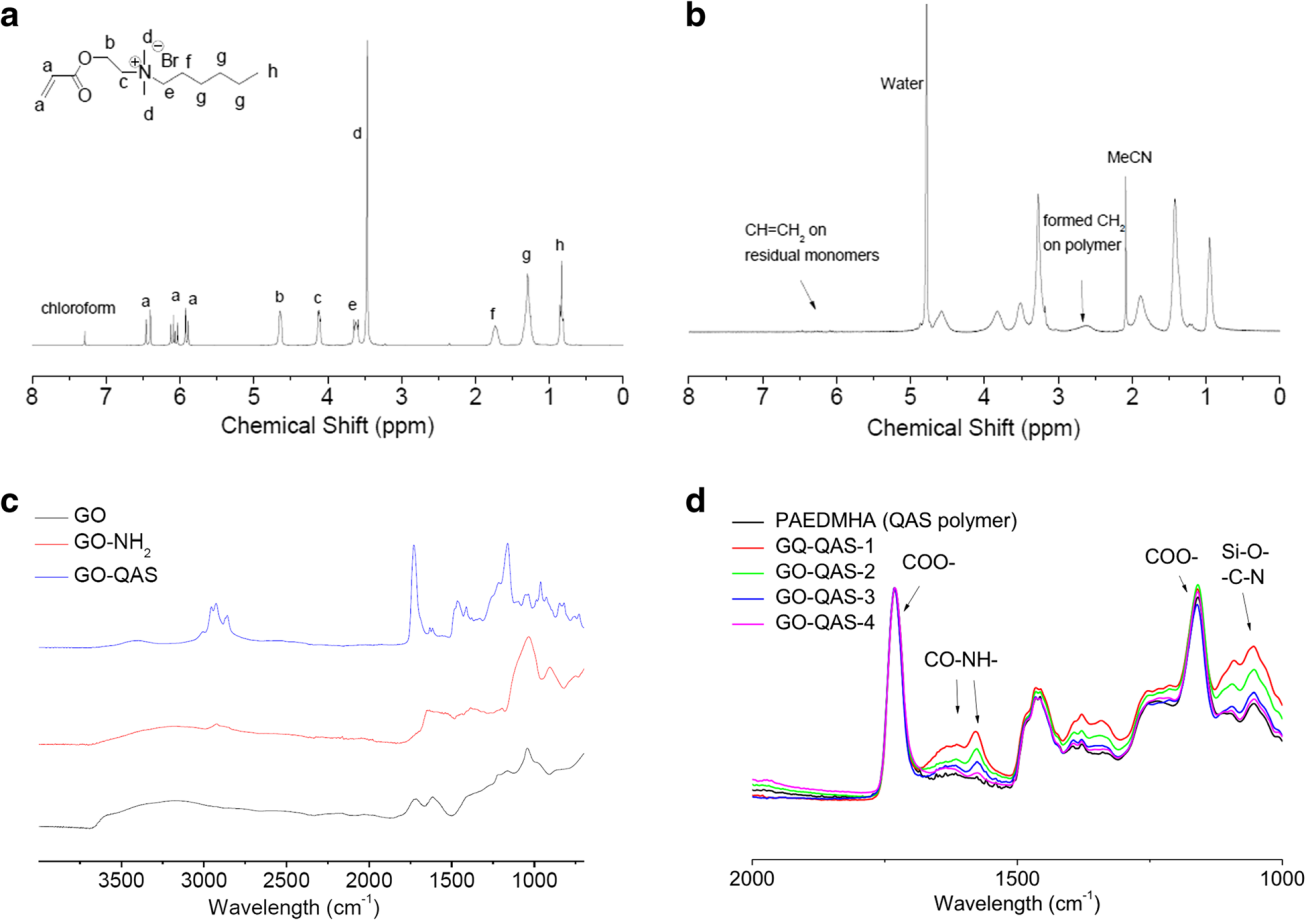
^1^H NMR spectra of **a** AEDMHA monomers and **b** their polymerized product PAEDMHS; FTIR-ATR spectra **c** of GO, GO-NH_2_, and GO-QAS. **d** FTIR-ATR spectra of GO-QAS nanocomposites with different QAS (PAEDMEHA) graft ratios and pure QAS polymer. (NMR: nuclear magnetic resonance; AEDMHA: (2-(acryloyloxy)ethyl)-N, N-dimethylhexa-ammonium bromide; PAEDMHS: polymerization of AEDMHA monomers; PAEDMEHA: RAFT polymerization of AEDMHA quaternary ammonium salt; FTIR-ATR: Fourier transform infrared spectroscopy- attenuated total reflection; GO: graphene oxide; GO-NH_2_: amines grafted graphene oxide; GO-QAS: graphene oxide-quaternary ammonium salt)

The synthetic route for antibacterial GO-QAS nanosheets is shown in Scheme 1. The quaternary ammonium polymers containing GO nanosheets were fabricated via four reaction steps. The quaternary ammonium monomer was prepared by quaternization of the hydrophobic compound 1-bromohexane with an acrylate monomer containing the tertiary amine 2-(dimethylamino) ethyl acrylate (DMAEA). The quaternary ammonium monomer AEDMHA was sequentially polymerized via the reversible addition-fragmentation chain transfer (RAFT) polymerization technique, which is versatile and has a high tolerance to functional groups. The monomers were initiated with a carboxylic acid-containing chain transfer agent (RAFT-COOH) to polymerize the carboxylic group end-capped QAS polymer chains. Their ^1^H NMR spectra are presented in Fig. 1a, b. Compared with the spectrum of monomers, the peaks at 5.90~6.40 ppm assigned to protons on C=C double bonds disappeared after polymerization, indicating successful polymerization. The broadened peaks of quaternary ammonium pendants also confirmed the formation of a polymeric configuration, and the broad peak at 2.55 ppm that appeared was attributed to the newly formed -CH–CH_2_- single bonds on the polymer backbone. The polymerization degree could be estimated by comparing the peak intensity of the three residual peaks at 5.90~6.40 ppm from the three protons on acrylates with the peak at 0.9 ppm attributed to the methyl groups on the pendant ends, and the number-average molecular weight (Mn) of the polymers was calculated as ~ 7400 Da, with a conversion rate of ~ 93%. To conjugate the carboxylic acid end-capped polymers, the GO nanosheet surface was decorated with amine groups via silanization with (3-aminopropyl)trimethoxysilane (APTMS). After silanization, the GO-NH_2_ suspension became aggregated and viscous, which may be attributed to ionic interactions between the introduced amines and carboxylic acids on the GO nanosheets. Based on the reaction yield, at least 0.89 mol APTMS/100 g GO (or 133 g APTMS/100 g GO) should have been grafted onto nanosheets. Finally, the carboxylic acid-capped quaternary ammonium polymers were covalently bonded to GO-NH_2_ nanosheets via an amidation reaction with EDC/NHS catalysis, and four groups of GO-QAS nanocomposites with different QAS graft ratios were prepared by varying the feeding ratios of the GO-NH_2_ and QAS polymers. After removal of the unconjugated polymers via dialysis and lyophilization, the graft ratios of QAS in GO-QAS nanosheets were estimated by calculating the product yields, and the loading ratios of QAS changed from 50 to 86.3 wt% of the overall solid content (see Additional file 1: Table S1). The obtained GO-QAS nanocomposites showed good dispersion stability in water.

According to the FTIR-ATR spectra in Fig. 1c, the broad peak at 3200~3700 cm^−1^ was from the hydroxyl groups on GO, while the broad peak at 2500~3200 cm^−1^ was attributed to O-H stretching of carboxylic acid groups on the GO surface. After silanization, the sharp peak that appeared at 2940 cm^−1^ was from alkyl -CH- stretching in aminopropyl groups, while the strong peak at 1050 cm^−1^ is likely due to Si-O bond stretching, which confirmed successful silanization of APTMS on GO. Based on the spectrum of GO-QAS, the strong peaks at 2970 and 2940 cm^−1^ were due to C-H stretching in the-CH_3_ and -CH_2_- groups of the quaternary ammonium polymers. Moreover, the sharp strong peak at 1730 cm^−1^ was assigned to C=O stretching of esters in polyacrylate, while another strong peak at 1170 cm^−1^ was from C-O stretching of esters, which demonstrated the conjugation of polymer chains onto GO nanosheets. The high peak intensity of the polymer functional groups suggested a high polymeric content in the GO-QAS nanocomposite. To further confirm the various QAS graft ratios, four GO-QAS groups and pure QAS polymer were characterized by FTIR-ATR. The sharp peak at 1730 cm^−1^ was only attributed to ester groups in QAS polymers, while the peak at 1050 cm^−1^ was from the overlap of the strong peak of Si-O on GO-NH_2_ nanosheets and the weak peak of C-N bonds on both GO-NH_2_ and QAS polymers, and therefore, the graft ratios of QAS onto GO-NH_2_ could be evaluated by comparing the relative peak intensities of these two regions. As shown in Fig. 1d, the peak intensity at 1730 cm^−1^ was normalized for all samples, and the absorption peak intensity at 1050 cm^−1^ became weaker from GO-QAS-1 to GO-QAS-4 sequentially, showing that GO-QAS-1 has the lowest QAS graft ratio and GO-QAS-4 has the highest graft ratio among the nanocomposites.

**Fig. 2:**
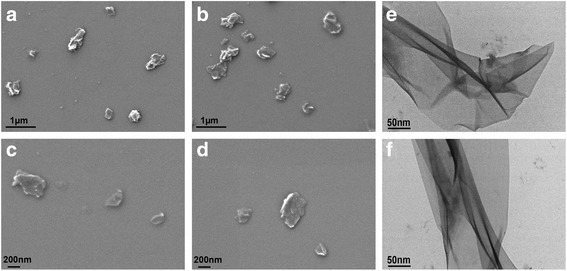
Scanning electron microscopy images of graphene oxide (GO) (**a**, **c**) and graphene oxide-quaternary ammonium salt (GO-QAS) (**b**, **d**). Transmission electron microscopy images of **e** GO and **f** GO-QAS

The surface morphologies of purchased GO and synthesized GO-QAS were explored by SEM and TEM. According to the SEM images in Fig. 2a–d, it was evident that GO possessed a number of closely stacked sheets with significantly wrinkled surfaces. In contrast, it seems that GO-QAS had relatively less closely stacked sheets and were better distributed than the GO nanosheets, which was attributed to the presence of QAS functioning as a cationic surfactant to facilitate dispersion, as shown in Figure S4 (see Additional file 1). Further and more distinct morphology confirmation was obtained from the TEM images in Fig. 2e–f. We found that both GO and GO-QAS exhibited a sheet-like morphology with high transparency. In addition, there were some wrinkles and folded regions in both the GO and GO-QAS nanosheets.

**Fig. 3:**
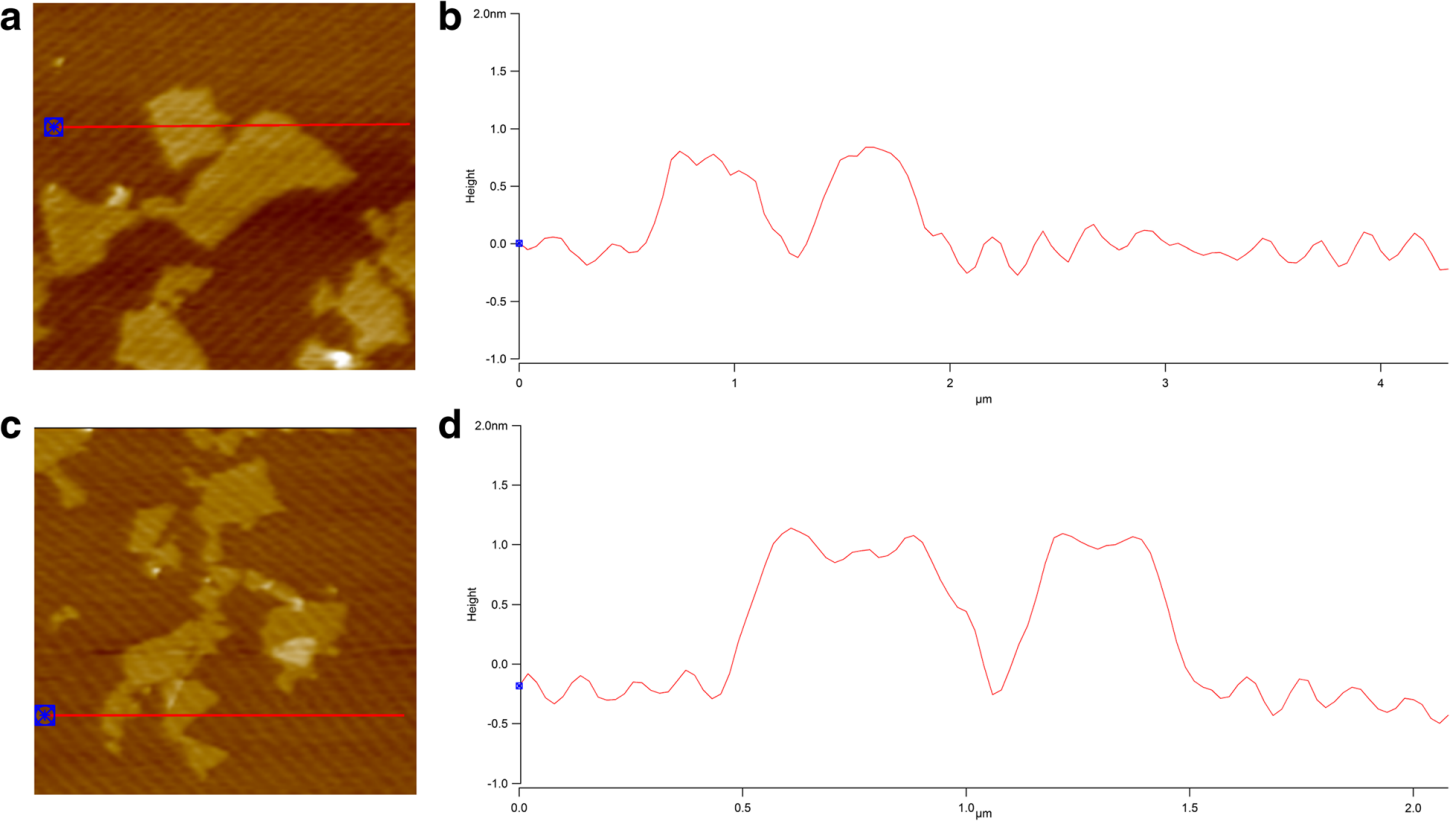
Atomic force microscopy results of graphene oxide (**a**, **b**) and graphene oxide-quaternary ammonium salt (**c**, **d**)

Based on the AFM height profiles shown in Fig. 3, both GO and GO-QAS were composed of monodispersed layers. The bare GO (Fig. 3b) sample exhibited a height of 0.8 nm, which was a characteristic of fully exfoliated single-layer GO [33]. However, GO-QAS (Fig. 3d) presented an approximately 1 nm height thickness, with a lateral size similar to that of GO. The increase in the height of GO-QAS was caused by the coverage of QAS on the basal plane of GO, which also indirectly indicated the formation of the GO-QAS nanocomposite.

**Fig. 4:**
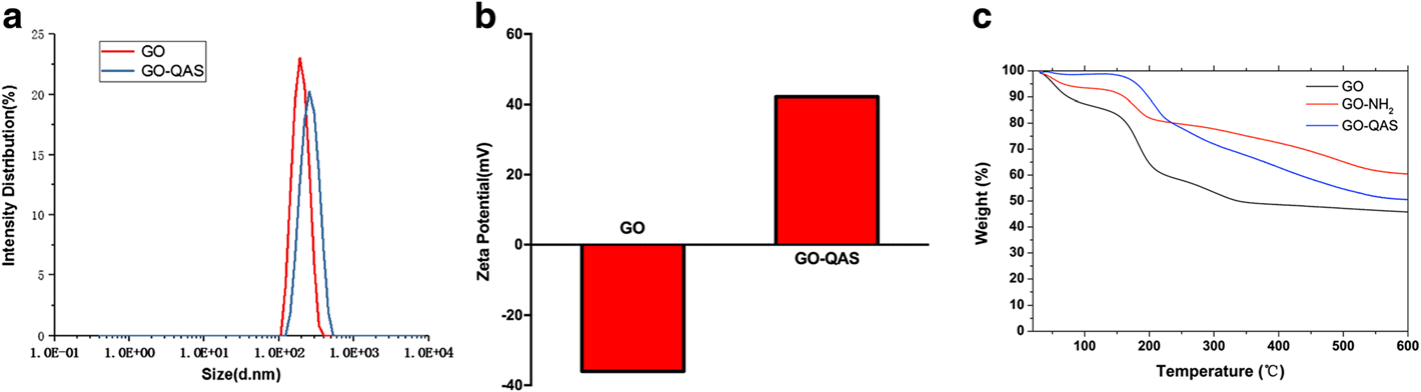
**a** Dynamic light scattering results of GO and GO-QAS. **b** Zeta potential results of GO and GO-QAS. **c** Thermogravimetric analysis results of GO, GO-NH_2_, and GO-QAS. (DLS: dynamic light scattering; TGA: thermogravimetric analysis; GO: graphene oxide; GO-NH_2_: amines grafted graphene oxide; GO-QAS: graphene oxide-quaternary ammonium salt)

The particle size distribution of GO and GO-QAS was characterized by DLS. According to the DLS results shown in Fig. 4a, we found that both GO and GO-QAS exhibited a wide size distribution, with the majority dispersed within a narrow range. The average particle size was 223 and 255 nm for GO and GO-QAS, respectively. The surface charge of the nanoparticles was characterized by zeta potential using DLS. As shown in Fig. 4b, the zeta potential of GO was − 36.03 mV, while the zeta potential of GO-QAS was 42.01 mV. The observed surface charge difference was due to the presence of positively charged QAS on GO nanosheets, indicating a successful synthesis of the GO-QAS nanocomposite.

The thermal stability of pre-dried GO, GO-NH_2_, and GO-QAS (GO-QAS-3) samples was characterized by thermogravimetric analysis, as presented in Fig. 4c. When the temperature was below 140 °C, the mass losses were 15.8, 7.8, and 1.4% for GO, GO-NH_2_, and GO-QAS (GO-QAS-3), respectively, due to water loss. Interestingly, the water loss seemed to mainly be attributed to the GO nanosheet binding interaction and was proportional to the GO weight fractions in the nanocomposites. By calculation, the GO-QAS nanocomposites contained 8.9 wt% GO, 9.2 wt% APTMS, and 81.9 wt% QAS, which was in line with the weight fractions based on reaction yields. At 150~200 °C, graphene oxide began to degrade due to deoxygenation of the carboxylic acid and hydroxyl groups. GO and GO-NH_2_ had ~ 20 and ~ 10% mass loss, respectively, according to their GO content. GO-QAS had ~ 9% mass loss not only because of deoxygenation but also due to Hofmann degradation of quaternary ammonium salts, and therefore, GO-QAS could maintain thermal stability below 150 °C.

### *In vitro* antibacterial activity of GO-QAS nanosheets

#### Optimization of the antibacterial activity of GO-QAS by modulating the QAS graft ratio

**Fig. 5:**
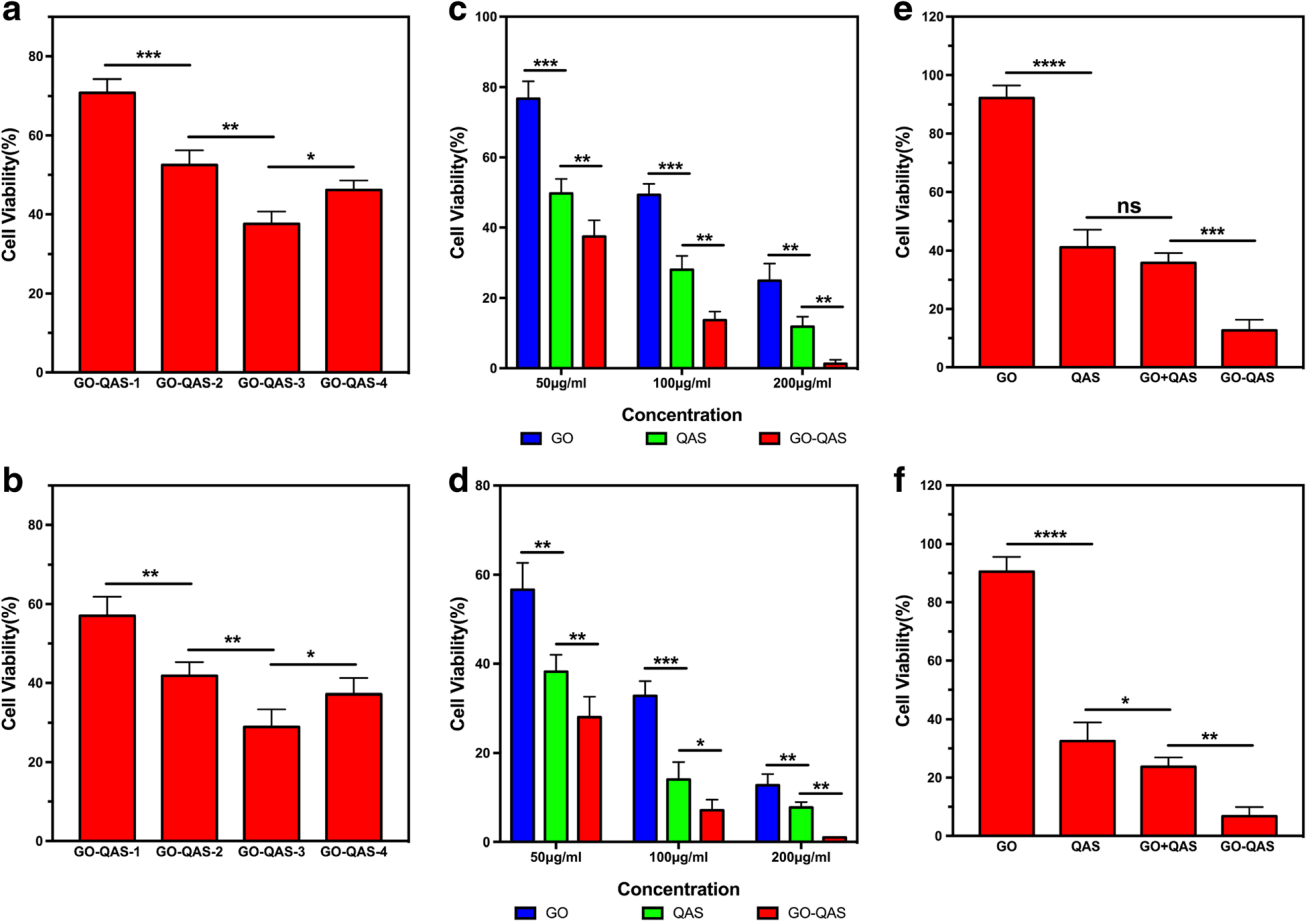
Antibacterial activity of GO-QAS with different QAS graft ratios against **a** *Escherichia coli* and **b** *Staphylococcus aureus*. Concentration-dependent antibacterial activity of GO-QAS with optimal QAS graft ratio against **c** *Escherichia coli* and **d** *Staphylococcus aureus*. Synergistic antibacterial effect of GO-QAS against **e** *Escherichia coli* and **f** *Staphylococcus aureus*. (ns represents *p* > 0.05; *p < 0.05, **p < 0.01, ***p < 0.001, ****p < 0.0001). (GO-QAS: graphene oxide-quaternary ammonium salt; QAS: quaternary ammonium salt; *S. aureus*: *Staphylococcus aureus*; *E. coli*: *Escherichia coli*)

To optimize the QAS graft ratio so that GO-QAS could exhibit the highest possible antibacterial activity, we tailored the overall graft ratios of QAS polymers on GO nanosheets to evaluate the antibacterial capacity of four GO-QAS (GO-QAS 1, 2, 3, and 4) nanocomposites at 50 μg/mL with different quaternary ammonium salt levels of 50, 69.7, 81.3, and 86.3 wt%, respectively (see Additional file 1: Table S1). As shown in Fig. 5a–b, an increased QAS graft ratio enhanced the antibacterial activity of GO-QAS when the QAS mass fraction in GO-QAS was up to 81.3% (GO-QAS-3), but a continuously increased QAS graft ratio led to a drop in antibacterial performance. Among the four GO-QAS nanosheet groups, GO-QAS-3 showed the highest antibacterial activity against both *E. coli* and *S. aureus*. Therefore, GO-QAS-3 (the optimized GO-QAS) was utilized in the following experiments.

#### Antibacterial activity of optimized GO-QAS nanosheets at different concentrations

##### Agar diffusion test

The antibacterial activity of optimized GO-QAS nanosheets was qualitatively analyzed and compared with GO by observing the transparent halo around the well (see Additional file1: Figure S1). It was found that GO-QAS at a concentration higher than 10 μg/mL had obviously visible bactericidal activity against both gram-positive and gram-negative bacteria. In addition, the inhibition zone for gram-positive bacteria was much clearer and larger than that for gram-negative bacteria. Based on the agar diffusion test results, we chose GO-QAS dispersion concentrations of 50, 100, and 200 μg/mL to carry out the following experiments.

##### Plate count method

The bactericidal effect of GO, QAS, and GO-QAS dispersions on gram-positive and gram-negative bacteria was quantitatively evaluated by counting the number of colony forming units (CFU) on an agar plate (Fig. 5c–d). The results showed that GO, QAS, and GO-QAS could significantly eradicate both gram-positive and gram-negative bacteria. The cell viability decreased gradually as the concentration increased, which was demonstrated by the sequentially decreasing number of CFU on the LB plates (see Additional file1: Figure S3). GO-QAS at 200 μg/mL almost completely killed both gram-positive and gram-negative bacteria, while GO and QAS at the highest concentration caused approximately 87% and 92% cell death for *S. aureus* and 75% and 88% cell death for *E. coli*, respectively.

##### Bacterial live/dead viability assay

**Fig. 6:**
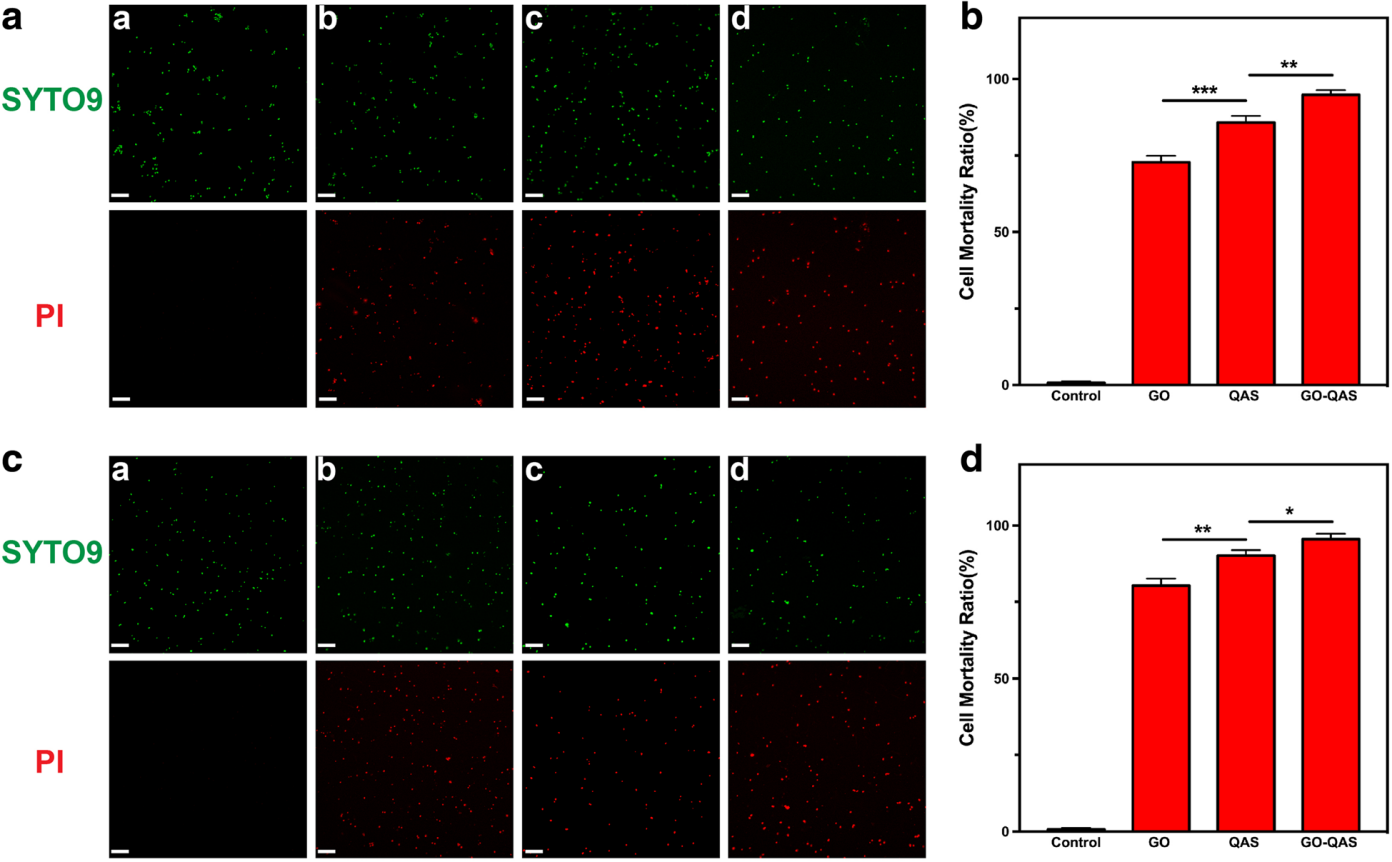
Fluorescence microscope images of **a** *Escherichia coli* and **c** *Staphylococcus aureus* in (a) control, (b) GO, (c) QAS, and (d) GO-QAS groups after being stained with SYTO 9 and PI (scale bars 20 μm). The cells were observed directly at ×100 magnification by laser confocal microscopy. Red stands for dead cells and green stands for total cells. Cell mortality ratio of **b** *Escherichia coli* and **d** *Staphylococcus aureus* after GO, QAS, and GO-QAS incubation. (*p < 0.05, **p < 0.01, ***p < 0.001). (GO: graphene oxide; QAS: quaternary ammonium salt; GO-QAS: graphene oxide-quaternary ammonium salt; PI: propidium iodide; *S. aureus*: *Staphylococcus aureus*; *E. coli*: *Escherichia coli*)

To demonstrate the reliability of the GO, QAS, and GO-QAS antibacterial activity results obtained using the plate count method, a live/dead viability assay was performed to further evaluate the antimicrobial activity of GO, QAS, and GO-QAS at 200 μg/mL. Typically, SYTO 9 dye (green) stains both live and dead cells, while PI (red) only stains dead cells with compromised membranes. The cell mortality ratio % is expressed as the number of dead cells (red)/the number of total cells (green)×100%. As shown in Fig. 6, bacteria in the control group were all stained green, while some bacteria incubated with GO, QAS, and GO-QAS were stained red. The results showed that GO-QAS at 200 μg/mL could kill 95.5% of gram-positive and 95% of gram-negative bacteria, while GO and QAS could kill 80% and 90% of gram-positive and 73 and 85% of gram-negative bacteria, respectively.

#### Synergistic antibacterial effect of GO-QAS

Based on the results shown in Table S1 (see Additional file 1), the GO and QAS content in optimized GO-QAS (GO-QAS-3) was 8% and 81.3%, and thus, 100 μg/mL of GO-QAS suspension contained 8 μg/mL GO and 81.3 μg/mL QAS. To further demonstrate that the significantly enhanced antibacterial activity of GO-QAS nanocomposite was attributed to a synergistic antibacterial effect of GO and QAS, instead of merely an additive effect of the two constituents mixed together, bacterial cells were treated with 8 μg/mL GO, 81.3 μg/mL QAS, a mixture of 8 μg/mL GO and 81.3 μg/mL QAS, or 100 μg/mL of GO-QAS. As shown in Fig. 5e–f, the losses in *E. coli* cell viability were only 7.8%, 58.8%, and 61.8% with GO, QAS, and the mixture of GO and QAS, respectively. For *S. aureus*, the results were 9.5%, 67.5%, and 74.6%, respectively. By comparison, the losses in *E. coli* and *S. aureus* viability were 87.4% and 93.2% with GO-QAS, respectively. Therefore, the combined antibacterial activity of GO-QAS was not simply the additive effect of the two constituents, but rather attributed to a synergistic antibacterial effect.

#### Antibacterial mechanisms of GO-QAS nanosheets

##### Cell morphology observation via SEM

**Fig. 7:**
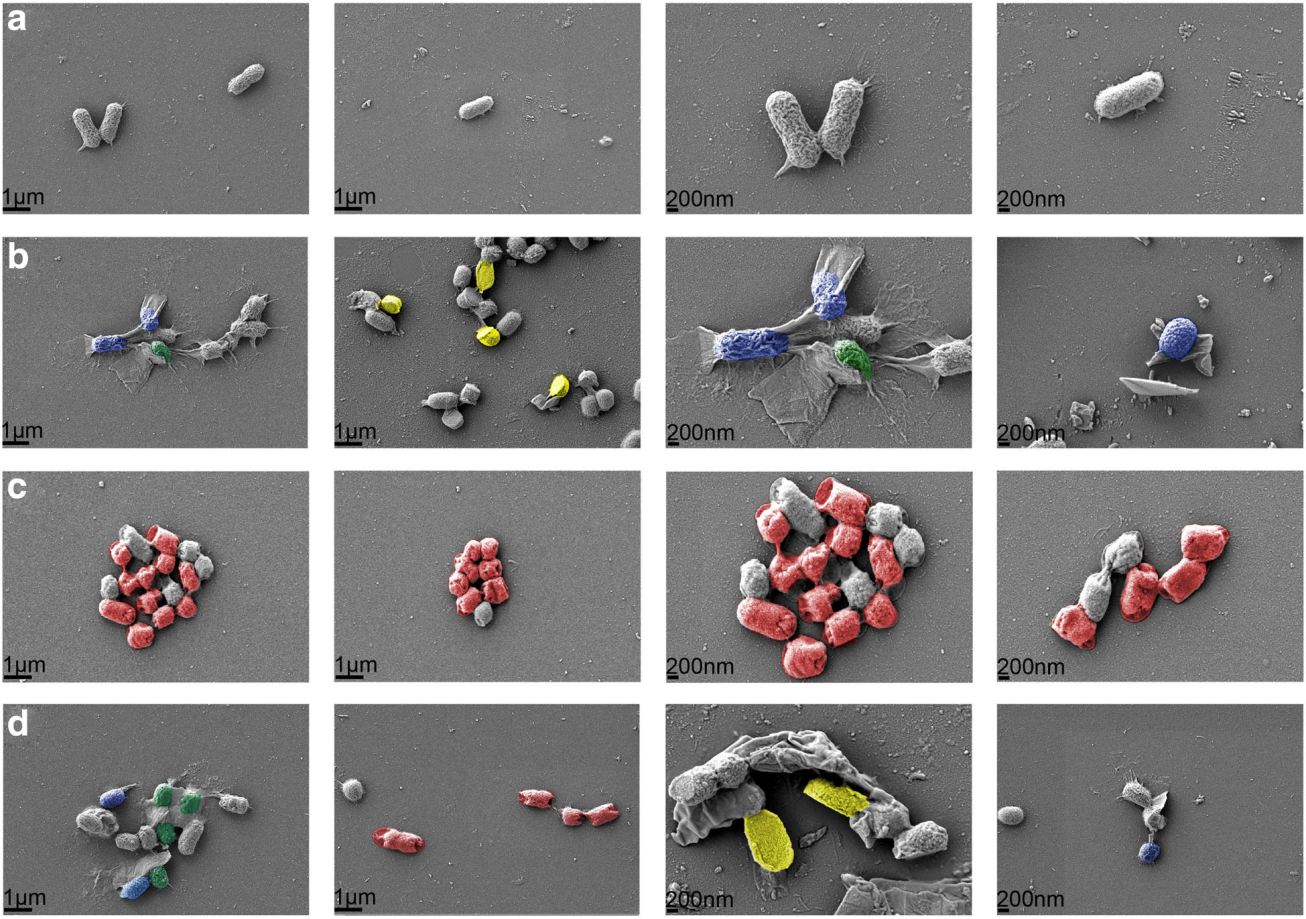
Scanning electron microscopy images of *Escherichia coli* cells treated **a** without nanocomposite, **b** with graphene oxide (GO) suspensions, **c** with quaternary ammonium salt (QAS) suspensions, and **d** with GO-QAS suspensions, respectively. Bacteria stained in green color represent nanosheets wrapping/trapping bacteria. Bacteria stained in blue color indicate nanosheets inserting into bacteria membrane. Bacteria stained in red color denote bacteria membrane perforated by nanocomposite with pores formed on the cell membrane. Bacteria stained in yellow color indicate bacteria split into two halves by nanosheets

To investigate the bacterial morphology changes after GO-QAS treatment, SEM was applied to observe *E. coli* morphology after exposure to nanosheets for 2 h. According to the SEM results shown in Fig. 7, *E. coli* without nanosheet treatment showed a typical rod-shaped morphology, with intact and smooth cell walls. However, after being treated with GO, QAS, and GO-QAS, the majority of bacteria lost their cellular integrity, and bacteria length tended to shorten, with the bacterial surface becoming rough and wrinkled. Interestingly, we found that *E. coli* were wrapped up or stabbed by nanosheets and cell walls collapsed after GO and GO-QAS treatments (bacteria stained green and blue in Fig. 7). Such results were consistent with previous reports on the antibacterial mechanism of GO [33], indicating that the wrapping/trapping and incision mechanisms were shared by both GO and GO-QAS. Notably, the physical cleavage force was so strong that we observed many cells split into two halves (bacteria stained yellow in Fig. 7), which to the best of our knowledge has never been reported in previous literature. Additionally, there were some pores formed on the cell membrane after GO-QAS treatment (bacteria stained red in Fig. 7), which was similar to the morphology of *E. coli* after QAS treatment, but quite different from that after GO treatment.

##### Measurement of cytoplasmic DNA and RNA efflux

**Fig. 8:**
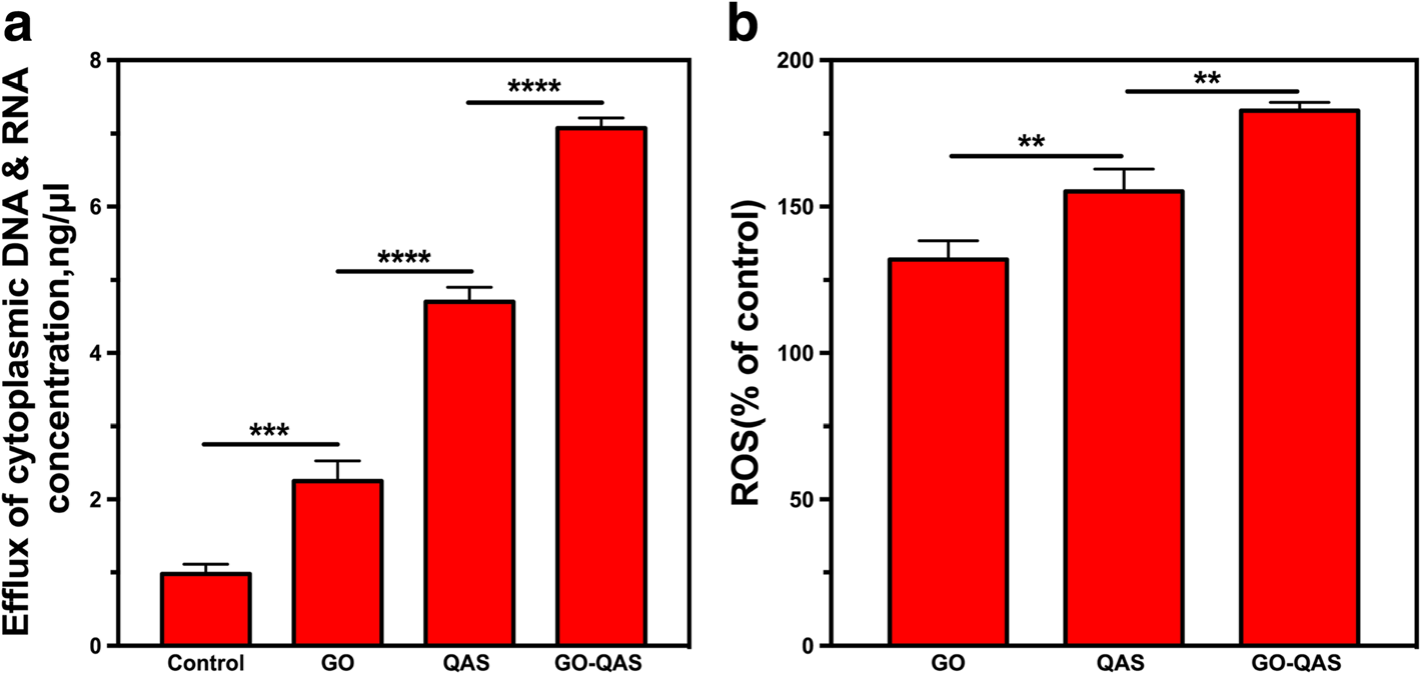
**a** The absorbance of efflux of cytoplasmic materials (DNA and RNA) at 260 nm after bacteria being incubated with GO, QAS, and GO-QAS at 200 μg/mL, respectively. **b** ROS generation after the bacteria suspensions were incubated with GO, QAS, and GO-QAS dispersions at 200 μg/mL concentration. (**p < 0.01, ***p < 0.001, ****p < 0.0001). (GO: graphene oxide; QAS: quaternary ammonium salt; GO-QAS: graphene oxide-quaternary ammonium salt; ROS: reactive oxygen species)

If the bacteria membrane was disrupted, the cytoplasmic contents, such as nucleic acids (DNA and RNA), would be released from the damaged bacteria and could be monitored in the supernatant via the 260 nm absorption. After bacteria were incubated with GO, QAS, and GO-QAS, the nucleic acid concentrations in the supernatant were measured (Fig. 8a). Compared with the control group, the nucleic acid concentrations were significantly higher after GO, QAS, and GO-QAS treatments. Such results further confirmed the bacteria membrane disruption observed with SEM. The efflux concentrations of cytoplasmic nucleic acids were 2.25, 4.7, and 7.1 ng/μL with GO, QAS, and GO-QAS, respectively. The nucleic acid concentration in the GO-QAS group was significantly higher than those in the GO and QAS groups. These results indicated that the membrane disruption ability of GO-QAS was stronger than that of GO and QAS, likely due to the synergistic effect of the grafted QAS and GO nanosheets.

##### Reactive oxygen species detection

To further investigate whether the antimicrobial mechanism of GO-QAS involved oxidative stress induction, the ROS content in bacteria after nanosheet treatment was measured using DCFH-DA. Briefly, DCFH-DA is a non-fluorescent chemical compound and can freely penetrate the cell membrane. After entering the cell, it is hydrolyzed to DCFH by intracellular esterase and becomes unable to permeate the cell membrane. Intracellular ROS oxidize the non-fluorescent DCFH to fluorescent DCF, which can be measured at a 488-nm excitation wavelength and a 525-nm emission wavelength. Thus, the intracellular ROS level is indicated by the increase in fluorescence intensity. As shown in Fig. 8b, the ROS level was approximately 1.3-fold, 1.5-fold, and 1.8-fold higher than that of the control after GO, QAS, and GO-QAS treatments, respectively.

#### Antimicrobial property of GO-QAS against multidrug-resistant bacteria

The antimicrobial property of GO-QAS against MRSA and MDR-AB is shown in Additional file 1: Figure S2. The results illustrate that GO-QAS can significantly inhibit multidrug-resistant bacteria growth and eradicate both MRSA and MDR-AB. Compared with conventional antibiotics (penicillin and streptomycin), GO-QAS possessed more powerful antimicrobial activity against multidrug-resistant bacteria. In addition, we found that MRSA was more sensitive to GO-QAS than MDR-AB.

### *In vitro* cytotoxicity and hemocompatibility study

#### Cell viability test

**Fig. 9:**
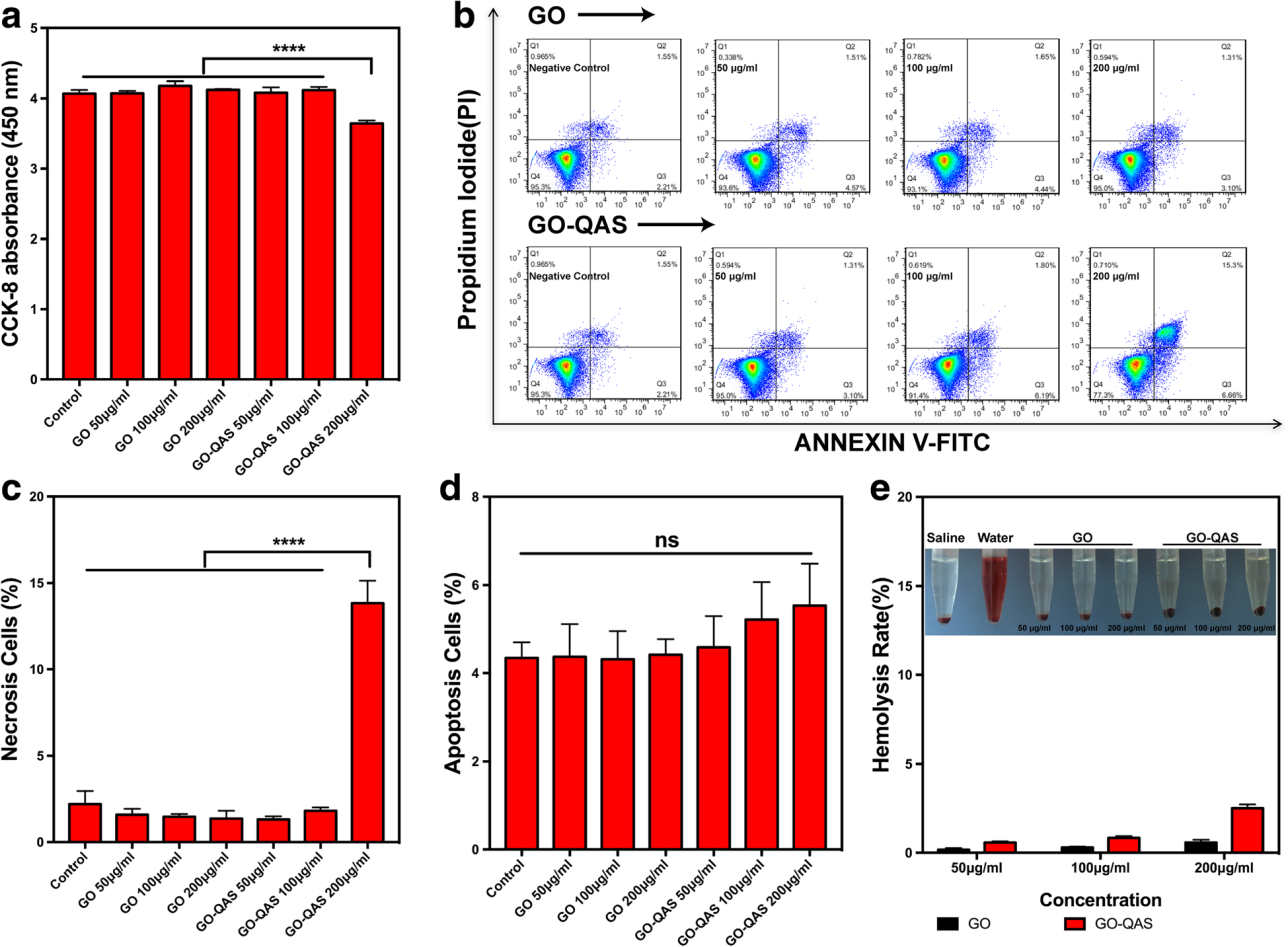
**a** Cell viability measured using CCK-8 assay after cells being incubated with different concentrations of GO and GO-QAS suspensions for 24 h. **b** FACS results showing cell apoptosis and necrosis distribution after GO and GO-QAS incubation based on the Annexin V–FITC and PI assay. Statistical analysis of **c** necrosis cells ratio and **d** apoptosis cells ratio. **e** Hemocompatibility of GO-QAS at different concentrations. (ns represents *p* > 0.05; ****p < 0.0001). (CCK-8: cell counting kit-8; GO: graphene oxide; GO-QAS: graphene oxide-quaternary ammonium salt; FACS: fluorescence-activated cell sorting; PI: propidium iodide)

The cytotoxicity of nanosheets to mammalian cells was evaluated using CCK-8 assays to measure the viability of HaCaT cells exposed to different concentrations of samples for 24 h (Fig. 9a). The results revealed that GO exhibited no obvious cytotoxicity to HaCaT cells even at the high concentration of 200 μg/mL, and GO-QAS at concentrations lower than 200 μg/mL showed no obvious cytotoxicity. However, GO-QAS at 200 μg/mL exhibited slight cytotoxicity towards HaCaT cells, evidenced by the cell viability decrease to approximately 90% after 24 h of incubation, which indicated that the cytotoxicity of GO-QAS was mainly caused by the grafted QAS group at high concentrations.

#### Flow cytometry apoptosis assay

To further determine the cytotoxicity of GO-QAS towards mammalian cells, we employed an Annexin V-FITC apoptosis detection kit to detect the ratio of apoptotic and necrotic cells after incubation with different concentrations of nanocomposites (Fig. 9b–d). The flow cytometry apoptosis assay results showed that there was no significant difference in the percentage of necrotic cells between the GO and GO-QAS groups at concentrations lower than 200 μg/mL. However, GO-QAS at 200 μg/mL induced a significantly higher ratio of necrotic cells (Fig. 9c), which corroborated the results obtained with the CCK-8 assays and inverted phase contrast microscopy. In addition, it was intriguing to find that there was no significant difference in the ratio of apoptotic cells (Fig. 9d) induced by GO and GO-QAS.

#### Hemolysis assay

As shown in Fig. 9e, the hemolysis induction rates of GO and GO-QAS nanosheets at different concentrations were significantly lower than 5%, indicating that both GO and GO-QAS (at 0–200 μg/mL concentrations) exhibited good hemocompatibility. Such result was further evident from the optical images (Fig. 9e, inset). In addition, the hemolysis rate in the GO-QAS group was slightly higher than that in the GO group at 200 μg/mL, which was in line with the cytotoxicity test results mentioned above. Therefore, GO-QAS at concentrations lower than 200 μg/mL could be identified as a biocompatible nanocomposite with no obvious cytotoxicity to mammalian cells or tendency to hemolyze erythrocytes.

### *In vivo* biosafety evaluation

**Fig. 10:**
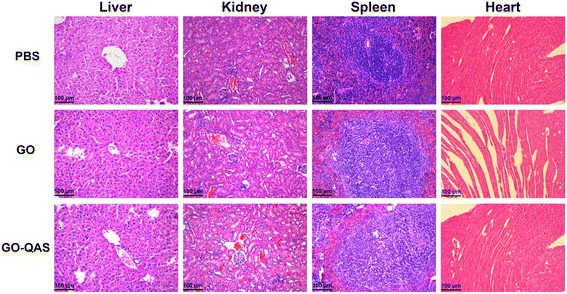
*In vivo* evaluation of the toxicity of GO and GO-QAS to major organs (liver, kidney, spleen, and heart) 7 days after intravenous administration. (PBS: phosphate buffer; GO: graphene oxide; GO-QAS: graphene oxide-quaternary ammonium salt)

To confirm the *in vitro* biocompatibility findings, the *in vivo* biosafety of GO and GO-QAS was determined. The *in vivo* toxicity of nanosheets was evaluated through intravenous administration. As shown in Fig. 10, compared with the control group, it was apparent that there was no obvious cellular swelling, necrosis, hemorrhage, or congestion observed in major organs 7 days after GO and GO-QAS injection at 100 μg/mL concentration, which indicated that GO and GO-QAS at 100 μg/mL were biocompatible. Combined with the *in vitro* cytotoxicity and hemolysis experiment results, GO-QAS dispersion at 100 μg/mL could be applied for further *in vivo* antimicrobial evaluation.

### *In vivo* antimicrobial evaluation

**Fig. 11:**
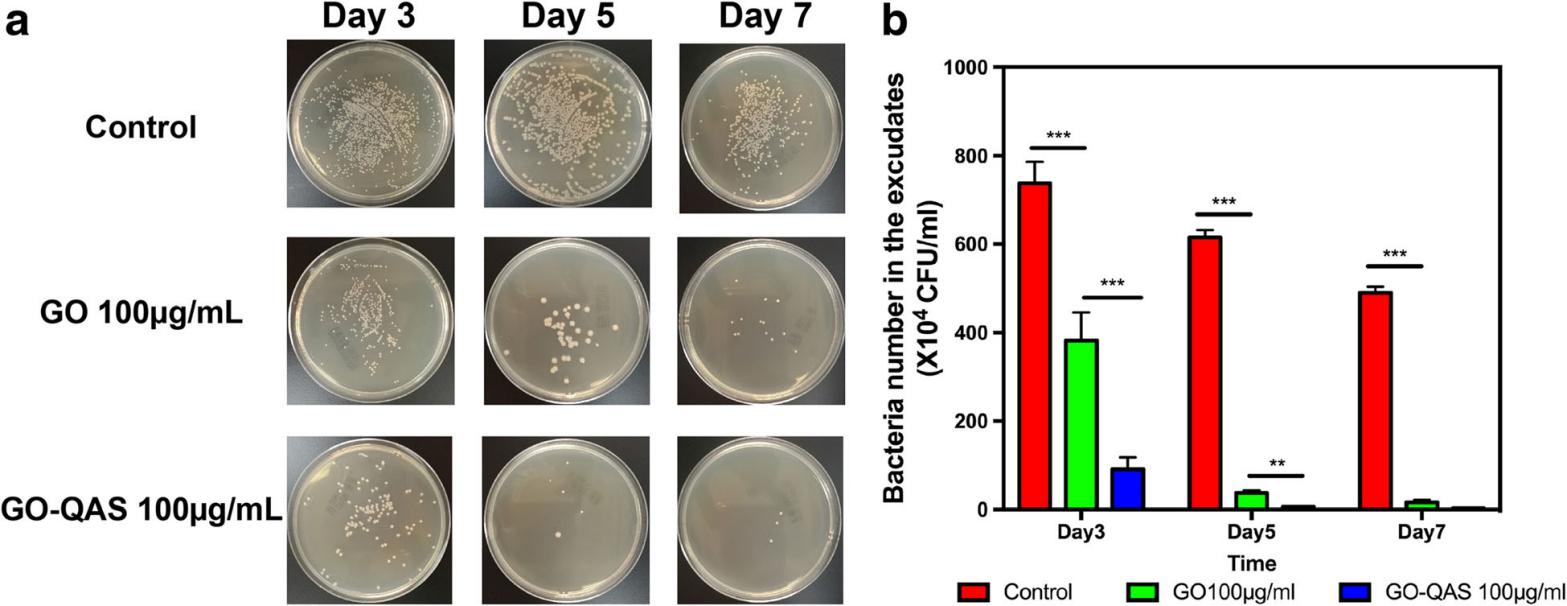
*In vivo* antimicrobial evaluation of GO-QAS. **a** The representative plate counting results of bacteria from the infected wound. **b** Quantitative statistics of the number of bacteria in the wound exudates on day 3, 5, and 7 post-infection. (*p < 0.05, **p < 0.01, ***p < 0.001). (GO: graphene oxide; GO-QAS: graphene oxide-quaternary ammonium salt; CFU/mL: colony-forming units per milliliter)

As shown in Fig. 11, for all three groups, the number of bacteria in the wound was the highest on the third day post-infection and then decreased gradually with time. On day 3 post-infection, the number of bacteria in the control group was approximately twofold higher than that in the GO group and eightfold higher than that in the GO-QAS group. Then, the number of bacteria in the GO and GO-QAS groups decreased on day 5 and day 7. Compared with the GO group, the GO-QAS group had the lowest number of bacteria in the wound throughout the experiment, which indicated that the antibacterial activity of GO-QAS was stronger than that of GO *in vivo*.

### *In vivo* evaluation of wound healing induced by GO-QAS nanosheets

**Fig. 12:**
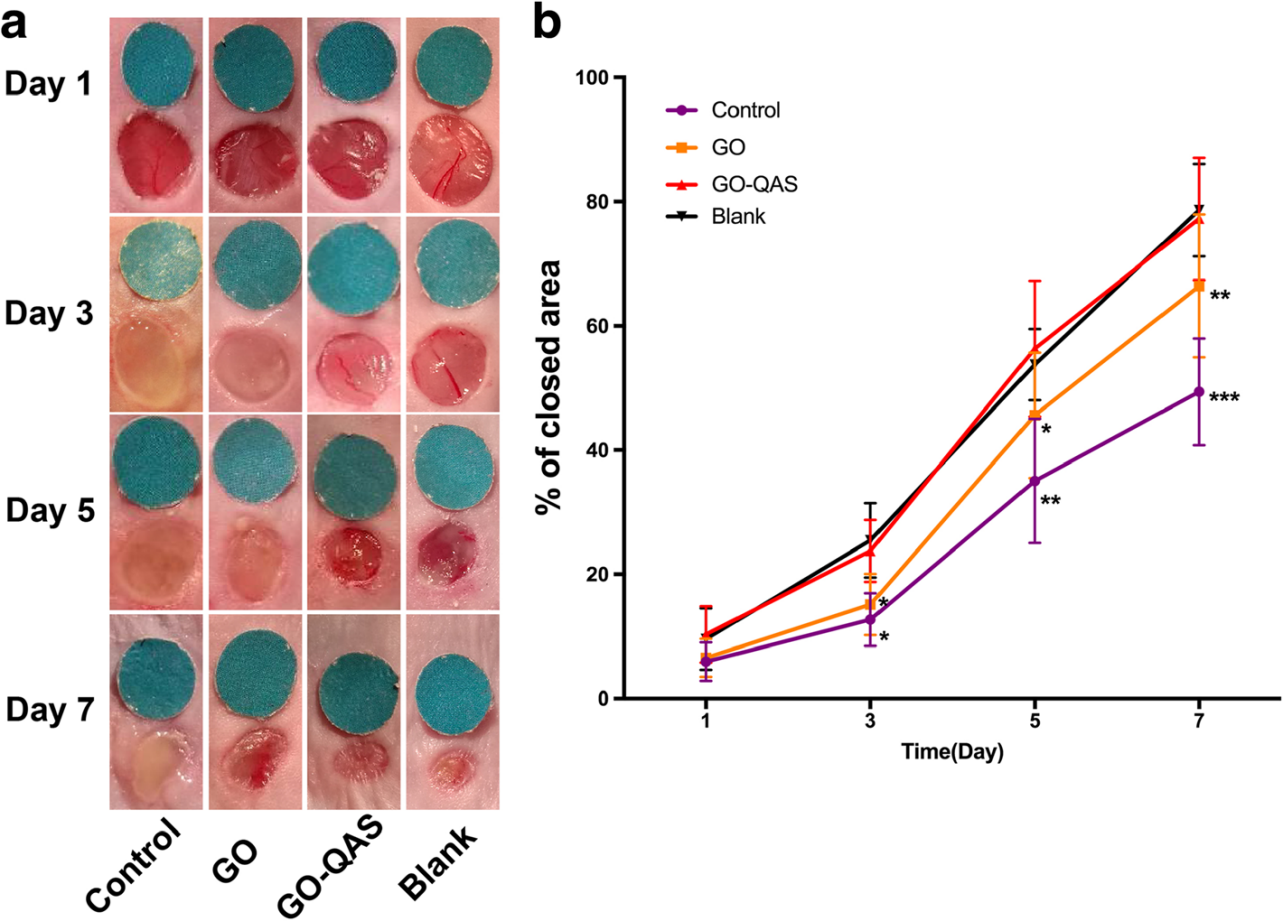
The effect of GO-QAS on infected wound healing. **a** The representative macroscopic appearance of wound from the blank, control, GO, and GO-QAS groups. **b** The area of wound closure at different time points. A 6-mm-diameter standard disc was used as the reference when taking photos. The values are shown as the mean ± SD (*n* = 10). Asterisks indicated the difference between each group and GO-QAS group. (*p < 0.05, **p < 0.01, ***p < 0.001). (GO: graphene oxide; GO-QAS: graphene oxide-quaternary ammonium salt)

The wound healing rate is shown in Fig. 12. On day 3 post-infection, obvious thick yellow pus was observed on the wounds of the control and GO groups. In addition, the skin around the wound was red and swollen in the control and GO groups. However, no obvious purulent discharge was found in the blank and GO-QAS groups. Furthermore, the wound closure area in the blank and GO-QAS groups was significantly larger than that of the control and GO groups. On day 5 post-infection, purulent exudates still existed in the control and GO groups. On day 7 post-infection, the percentage of the wound closure area was 78.7%, 58.9%, 66.4%, and 77.2% in the blank, control, GO, and GO-QAS groups, respectively. No significant difference was found between the blank and GO-QAS groups on any of the days. The results indicated that GO-QAS accelerated the healing rate of infected wounds.

### Histological analysis of infected wounds treated with GO-QAS nanosheets

**Fig. 13:**
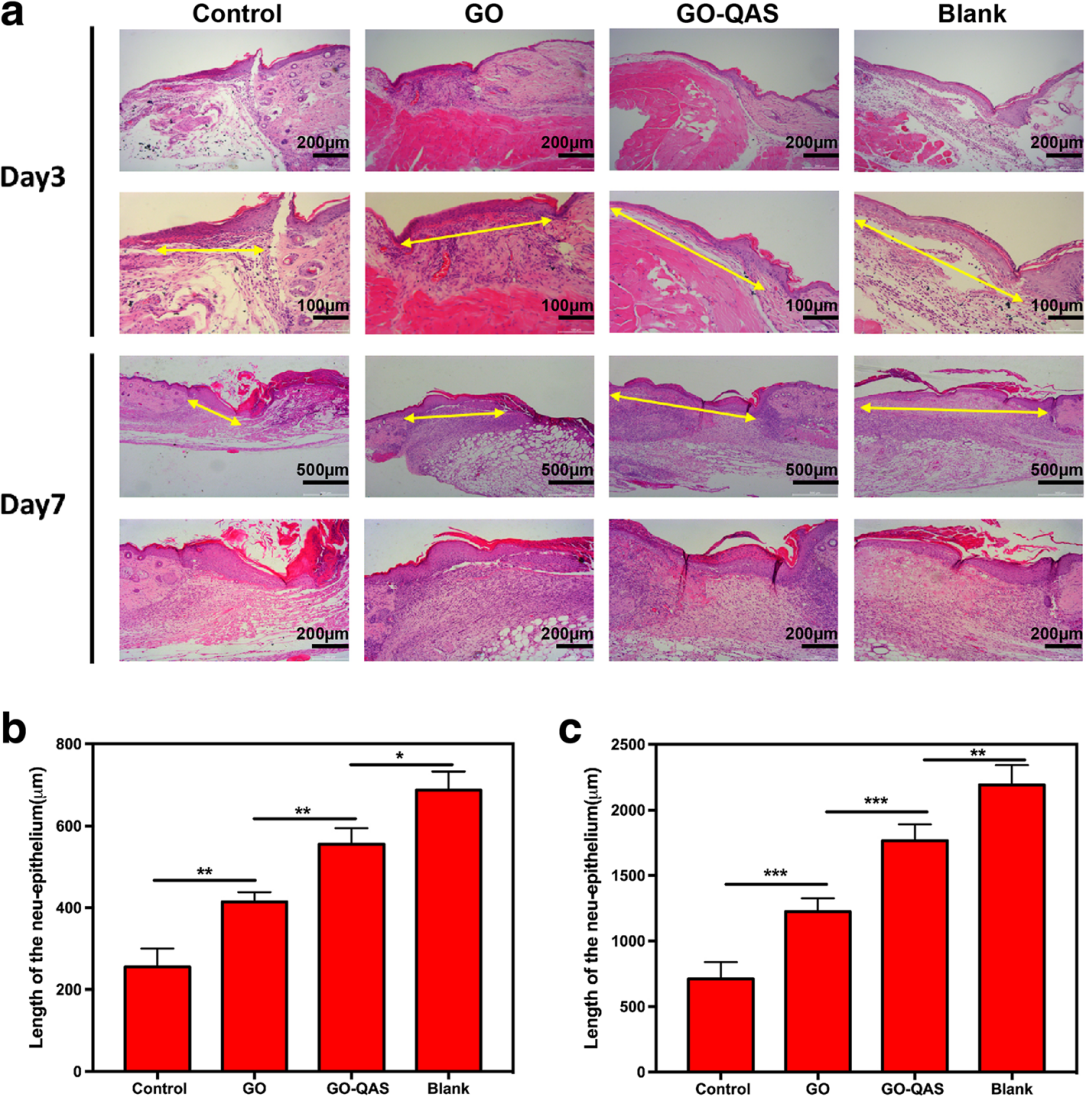
The effect of GO-QAS on re-epithelialization. **a** Representative histological images (with hematoxylin and eosin staining) of the length of the newly formed epidermis on day 3 and 7 post-infection in the control, GO, GO-QAS, and blank groups. The yellow double-headed arrows indicate the newly formed epidermis. **b** Measurement of the length of the newly regenerated epidermis on day 3. **c** Measurement of the length of the newly regenerated epidermis on day 7. (*p < 0.05, **p < 0.01, ***p < 0.001). (GO: graphene oxide; GO-QAS: graphene oxide-quaternary ammonium salt; H&E: hematoxylin and eosin)

**Fig. 14:**
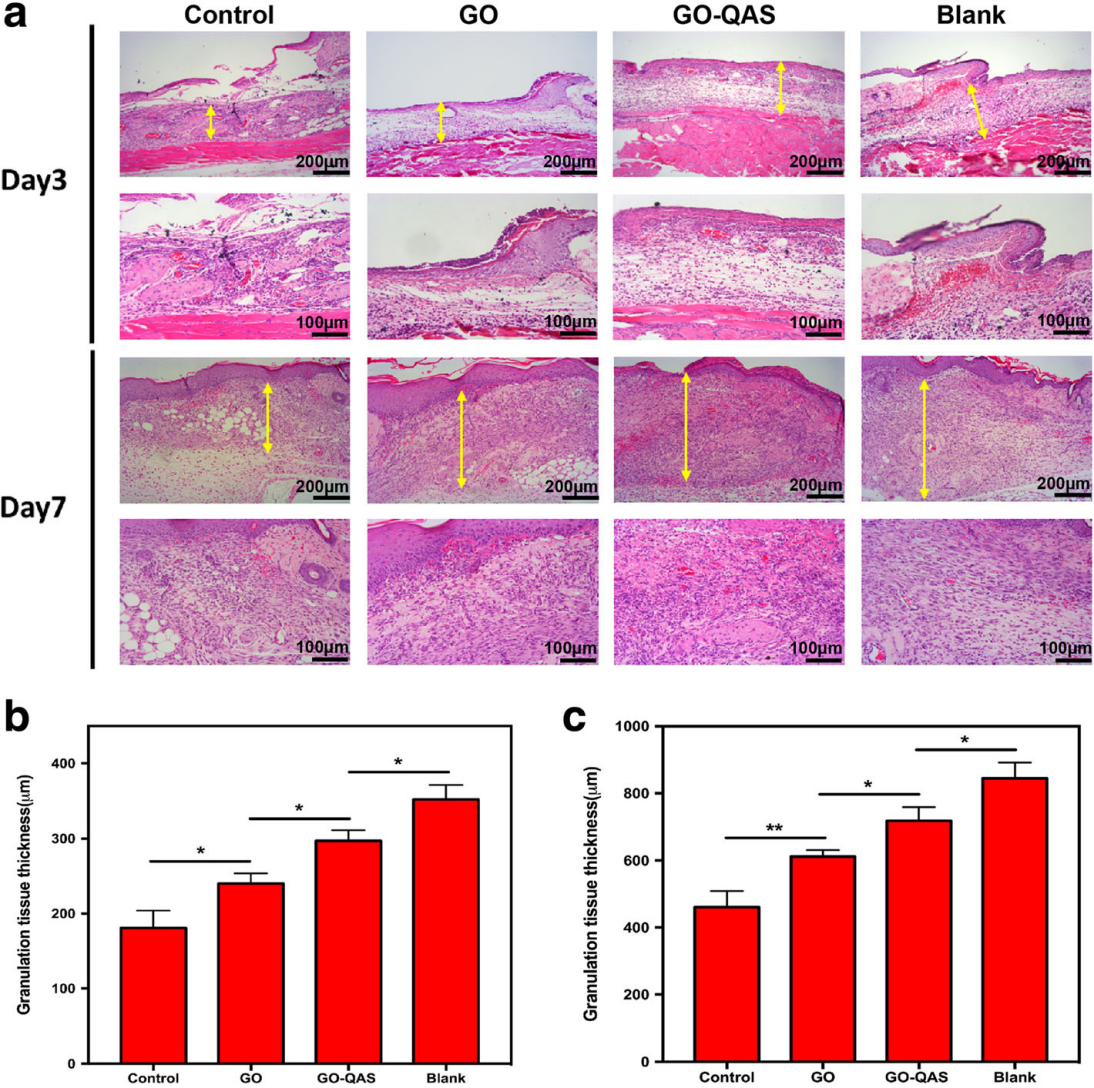
The effect of GO-QAS on granulation tissue formation. **a** Representative histological images (with hematoxylin and eosin staining) of the granulation tissue thickness on day 3 and 7 post-infection in the Control, GO, GO-QAS, and Blank groups. The yellow double-headed arrows indicate the granulation tissue. **b** Measurement of the granulation tissue thickness on day 3 post-infection. **c** Measurement of the granulation tissue thickness on day 7 post-infection. (*p < 0.05, **p < 0.01). (GO: Graphene oxide; GO-QAS: graphene oxide-quaternary ammonium salt; H&E: hematoxylin and eosin)

Based on H&E-stained sections, we found that the length of newly regenerated epidermis was significantly longer in the GO-QAS group than in the control and GO groups on days 3 and 7 post-infection, and there was little difference between the blank and GO-QAS groups (Fig. 13). Additionally, GO-QAS facilitated granulation tissue formation on days 3 and 7 compared with the control and GO groups. Similarly, there was little difference between the blank and GO-QAS groups in granulation tissue thickness (Fig. 14). These results indicated that GO-QAS could promote infected wound healing by accelerating re-epithelialization and granulation tissue formation.

## Discussion

Bacterial infection can aggravate wound deterioration, delay wound healing, and even cause sepsis and death [3]. However, the abuse of antibiotics in the past few decades has made it more difficult to eradicate pathogenic bacteria. Therefore, it is necessary to seek alternative materials to eradicate bacteria, such as nanomaterials. In recent years, GO nanosheets have been reported to exhibit excellent antibacterial activity [6]. However, graphene oxide nanosheets tend to aggregate in aqueous solutions in a layer by layer manner due to strong interplanar interactions, which limits their broad application [13]. In addition, there are also several reports claiming that GO possesses weak antimicrobial activity or even facilitates bacterial proliferation [17–19]. Thus, in this study, to confirm the antibacterial activity of GO and make it more stable in solution, a graphene oxide-quaternary ammonium salt (GO-QAS) nanocomposite was synthesized through amidation reactions of carboxylic group end-capped QAS polymers with primary amine-decorated GO. The grafted QAS groups served as cationic surfactants to maintain the stability of GO nanosheets in solution and enhanced the overall antibacterial activity.

The peak changes shown in the FTIR-ATR spectra (Fig. 1c) demonstrated successful synthesis of the GO-QAS nanocomposite. Then, the GO-QAS nanocomposite was characterized with SEM, TEM, AFM, DLS, zeta potential, and TGA. The SEM images showed that GO-QAS showed a sheet-like morphology with wrinkled surfaces (Fig. 2a–d), which was more distinct in the TEM images (Fig. 2e–f). The AFM and DLS measurements revealed that the thickness and particle size of GO-QAS were slightly larger than that of GO (Fig.3; Fig. 4a), which was caused by the coverage of QAS on the basal plane of GO and indirectly indicated the formation of the GO-QAS nanocomposite. The zeta potential difference between GO-QAS and GO was attributed to grafting of the positively charged QAS onto the negatively charged GO nanosheets (Fig. 4b), which further demonstrated successful synthesis of the GO-QAS nanocomposite. The TGA results demonstrated that the GO-QAS nanocomposite contained 8.9 wt% GO, 9.2 wt% APTMS, and 81.9 wt% QAS.

Since the QAS graft ratio in the GO-QAS polymer could significantly affect the antimicrobial properties, to determine the optimal QAS graft ratio at which the GO-QAS nanocomposite exhibited the strongest antibacterial activity, we investigated the antibacterial activity of GO-QAS with diverse QAS graft ratios. The cell viability results (Fig. 5a–b) showed that GO-QAS-3 (QAS mass fraction = 81.3%) exhibited the optimal QAS graft ratio, which also indicated that the antibacterial effect of GO-QAS was attributed not to GO or QAS alone but to a synergistic effect of GO and QAS. Since GO-QAS-3 showed the highest antibacterial activity, GO-QAS-3 (the optimized GO-QAS) was utilized in the following experiments.

After we determined the optimized QAS graft ratio, it was necessary for us to further detect the antibacterial properties of the optimized GO-QAS nanosheets. The results (see Additional file 1: Figure S1) showed that GO-QAS possessed visible bactericidal activity against both gram-positive and gram-negative bacteria. Interestingly, GO could not form an inhibition zone for either bacteria due to the poor diffusion property of GO on the agar plate, as previously reported [46], which might be caused by Van der Waals interactions between nanosheets. However, grafting of QAS onto GO nanosheets significantly increased the stability and dispersion properties of GO (see Additional file 1: Figure S4). It seems that the grafted QAS can weaken the interaction force between nanosheets. In addition, gram-positive bacteria were more sensitive to GO-QAS than gram-negative bacteria, since the inhibition zone was much clearer and larger, which might be because both GO and QAS were more effective against gram-positive bacteria [31]. A previous study reported that the external layers of the gram-negative bacterial cell wall, which is composed of lipopolysaccharides and proteins, can resist penetration of biocides. The cell wall of gram-positive bacteria, which is composed of peptidoglycan layers, can easily be penetrated [27]. The plate count results indicated that GO and QAS can kill both gram-positive and gram-negative bacteria but with limited antibacterial effect, while GO-QAS nanosheets significantly improved the killing efficiency (Fig. 5c–d). The bacterial live/dead viability assay results were not only in good accordance with the results obtained with the plate count method but also indicated that bacterial membranes were damaged after incubation with GO, QAS, and GO-QAS, which was further verified by observing cell morphology changes with SEM and measuring the efflux of cytoplasmic constituents (Fig. 6).

Since we have demonstrated the excellent antibacterial activity of GO-QAS, it was intriguing for us to detect whether the antibacterial activity of GO-QAS is attributed to a synergistic effect of GO and QAS or merely an additive effect of the two constituents mixed together. Bacteria were treated with GO, QAS, a mixture of GO and QAS, or GO-QAS. The antibacterial results (Fig. 5e–f) showed that GO-QAS exhibited higher antibacterial activity than the mixture of GO and QAS, indicating a synergistic antibacterial effect of GO and QAS. Such results could explain why there is an optimal QAS graft ratio at which GO-QAS exhibits the highest antibacterial activity. The mechanism of the synergistic antibacterial effect may be attributed to the following factors: (1) GO-QAS nanosheets have a much higher local QAS density/concentration on nanosheets compared with QAS solution and therefore could have much stronger antibacterial activity when in contact with bacteria. (2) QAS polymers could work as a surfactant to stabilize GO nanosheets in solution and prevent aggregation, which would improve the GO nanosheet antibacterial performance. (3) Positively charged GO-QAS nanosheets could be more easily adsorbed onto the negatively charged surface of the bacterial membrane through electrostatic interactions. Based on the antibacterial activity of GO, QAS, and GO-QAS, we could roughly calculate the contributions of each component to the antibacterial activity of GO-QAS using the following formula: antibacterial contribution % = individual antibacterial activity/antibacterial activity of GO-QAS ×100%. Therefore, for GO-QAS against *E. coli*, the antibacterial contributions of each part were 9% for GO and 67% for QAS. For *S. aureus*, the antibacterial contributions were 10% for GO and 72% for QAS.

Previous research has reported antibacterial activity of GO through membrane disruption and oxidative stress induction [11, 12] and the antimicrobial activity of QAS through cell membrane perturbation [25–27]. The bacterial live/dead assay results mentioned above indicated that the bacterial cell membrane was compromised after nanocomposite treatments. It was intriguing for us to determine whether GO-QAS shared the same and enhanced antibacterial mechanisms. Because gram-negative bacteria were more resistant to GO-QAS than gram-positive bacteria, we chose *E. coli* to carry out the following antibacterial mechanism experiments. SEM images of bacteria morphology (Fig. 7) indicated that GO-QAS exhibited the combined antibacterial mechanisms of GO and QAS. These results indicated a synergistic antibacterial effect of GO nanosheets and QAS, because not only did the GO-bacteria interaction contribute to the wrapping of the bacteria membrane with nanosheets, it created opportunities for full contact between grafted QAS and the cell membrane, thus increasing the local concentration of QAS on the bacteria membrane. Additionally, QAS facilitated the dispersion of GO nanosheets, thus making the interaction between GO and bacteria much easier. Moreover, although previous molecular dynamics simulations revealed that GO nanosheets could be spontaneously adsorbed onto a phospholipid membrane by the Van der Waals force [10], the positively charged GO-QAS nanosheets could be more easily adsorbed onto the negatively charged surface of the bacterial membrane through electrostatic interactions [47], thus causing more severe membrane damage. Bacterial membrane disruption was further demonstrated by measuring leakage of cytoplasmic material (Fig. 8a).

Oxidative stress is a highly recognized antibacterial mechanism of various carbon nanomaterials, such as graphene oxide [11, 12], fullerene [48], and carbon nanotubes (CNTs) [49]. Oxidative stress could subsequently disturb normal bacterial metabolism and cause protein denaturation, lipid peroxidation, and DNA damage, eventually leading to bacteria death [12]. The results (Fig. 8b) showed that both GO and GO-QAS could enhance the ROS level in bacteria, which corroborated previous literature reporting the antibacterial activity of graphene-based nanomaterials through ROS induction [7, 8]. In addition, QAS could also induce ROS production, which might be due to bacterial metabolism disruption after QAS enters the cytoplasm. Remarkably, GO-QAS induced higher ROS levels than GO at the same concentration, which indicated that the functional QAS group on GO could also induce ROS generation. These results revealed that GO-QAS can eradicate bacteria through oxidative stress induction.

After we demonstrated the excellent antibacterial activity of GO-QAS towards both gram-positive and gram-negative bacteria and revealed the non-specific antibacterial mechanisms of cell membrane disruption and oxidative stress induction, we decided to explore the antimicrobial property of GO-QAS against multidrug-resistant bacteria to determine whether such mechanisms worked against these bacteria. It was found that GO-QAS could significantly eradicate MRSA and MDR-AB (see Additional file 1: Figure S2), which indicated that the non-specific antimicrobial mechanisms (cell membrane disruption and oxidative stress induction) of GO-QAS could also be applicable to multidrug-resistant bacteria.

The excellent antimicrobial properties make it possible for GO-QAS to become a promising antibacterial agent. However, it was essential to demonstrate the biocompatibility of GO-QAS before it is utilized in the biomedical field. The cell viability test results (Fig. 9a) showed that GO-QAS at concentrations lower than 200 μg/mL showed no obvious cytotoxicity. These results were further demonstrated by the flow cytometry apoptosis assay (Fig. 9b–d). The results showed that GO-QAS at concentrations lower than 200 μg/mL caused no obvious cell apoptosis or necrosis. These results indicated that GO-QAS at concentrations lower than 200 μg/mL possessed good biocompatibility with mammalian cells.

Hemocompatibility is another important criterion to evaluate the biocompatibility of nanomaterials. Hemolysis should not occur when nanomaterials are used in blood-contact applications, such as applied on wounds. According to the YY/T0127.1 standard, a hemolysis rate higher than 5% is not acceptable for clinical use [50]. The hemolysis results (Fig. 9e) indicated that GO-QAS exhibited good hemocompatibility with erythrocytes. Then, we assessed the *in vivo* biosafety of GO-QAS via intravenous administration of GO-QAS to mice. The *in vivo* toxicity results (Fig. 10) showed that GO-QAS at 100 μg/mL was biocompatible. Combined with the *in vitro* cytotoxicity and hemolysis assay results, the GO-QAS dispersion at 100 μg/mL could be applied for further *in vivo* antimicrobial evaluation.

Since we demonstrated the antimicrobial activity of GO-QAS and detected the cytotoxicity of GO-QAS both *in vitro* and *in vivo*, it was imperative for us to detect the antibacterial efficiency of GO-QAS *in vivo*. For the *in vivo* antimicrobial evaluation, we chose GO-QAS at 100 μg/mL for the experiment because it caused no obvious toxicity to mammalian cells and organs according to the *in vitro* and *in vivo* toxicity tests results. The *in vivo* antibacterial results (Fig. 11) showed that compared with GO, GO-QAS still possessed excellent antibacterial activity *in vivo*, indicating the potential application of GO-QAS as an antibacterial agent to control wound infection.

Wound infection is one of the most threatening factors that contribute to prolonged wound healing. After demonstrating the significant antibacterial activity of GO-QAS *in vivo*, it was intriguing for us to further investigate whether topical application of GO-QAS could accelerate the infected wound healing process by eradicating pathogenic bacteria on wounds. The wound healing rate results showed that GO-QAS could significantly promote wound healing compared with the control and GO groups (Fig. 12). This result indicated that GO-QAS could significantly promote infected wound healing by eradicating pathogenic bacteria on wounds and maintaining an aseptic wound healing environment. These results were also in good accordance with other studies, which proposed that GO-containing wound dressings could promote wound healing by eradicating bacteria on wounds [8].

Because re-epithelialization and granulation tissue formation are two essential factors that contribute to wound healing [51], it was necessary for us to measure the length of the neo-epithelium and the granulation tissue thickness through histological analysis. The histological analysis revealed that compared with the control and GO groups, the length of newly regenerated epidermis and the thickness of granulation tissue were significantly greater in the GO-QAS group (Fig. 13 and Fig. 14). These results indicated that GO-QAS could promote infected wound healing by accelerating re-epithelialization and granulation tissue formation, which was attributed to the favorable antibacterial activity and biocompatibility of GO-QAS.

## Conclusions

The present study reported the synthesis of a novel antibacterial nanocomposite by grafting QAS onto GO nanosheets through surface modification and optimizing the QAS ratio. The as-prepared GO-QAS nanosheets exhibited proper biocompatibility both *in vitro* and *in vivo*. With synergistically enhanced antibacterial activity compared with pure GO, QAS, and the simple mixture of GO and QAS, the GO-QAS nanosheets exhibited highly effective antibacterial activity towards *S. aureus*, *E. coli*, and multidrug-resistant bacteria through bacterial membrane perturbation and oxidative stress induction. In addition, the GO-QAS nanocomposite maintained the natural and aseptic wound environment by eradicating pathogenic bacteria in wounds, which in turn accelerated the infected wound healing process by promoting re-epithelialization and granulation tissue formation. The overall results of this study indicated that the GO-QAS nanocomposite could be a promising antimicrobial agent for infected wound management and antibacterial wound dressing synthesis.

## Additional file

Additional file 1: **Table S1.** The conjugated GO-QAS nanocomposites reaction yields and estimated mass fraction of GO and QAS in the nanocomposites; **Figure S1.** Evaluation of antibacterial activity of GO and GO-QAS against *E. coli* and *S. aureus* by agar diffusion assay; **Figure S2.** Evaluation of the antimicrobial activity of GO-QAS against MRSA and MDR-AB; **Figure S3.** Plate count method results of *E. coli* and *S. aureus* after incubation with different concentrations of GO, QAS, and GO-QAS dispersions. **Figure S4.** Photograph of GO and GO-QAS nanosheets dispersed in different aqueous solutions without sonication. An additional file shows these data [see Additional file 1]. (DOC 10857 kb)

## Abbreviations

CCK-8: Cell counting kit-8
CFU/mL: Colony-forming units per milliliter
*E. coli*: *Escherichia coli*
GO: Graphene oxide
GO-QAS: Graphene oxide-quaternary ammonium salt
HaCaT: Human immortalized keratinocyte
LB: Luria-Bertani
MDR-AB: Multidrug-resistant *Acinetobacter baumanii*
MRSA: Methicillin-resistant *Staphylococcus aureus*
OD: Optical density
QAS: Quaternary ammonium salt
ROS: Reactive oxygen species
*S. aureus*: *Staphylococcus aureus*
