# Supplementary material for: Synthesis of graphene oxide-quaternary ammonium nanocomposite with synergistic antibacterial activity to promote infected wound healing
Source: Burns Trauma. 2018 May 21;6:16. doi: 10.1186/s41038-018-0115-2 (PMC5961493; doi:10.1186/s41038-018-0115-2)
Supplement: Supplementary file 1 — Table S1. The conjugated GO-QAS nanocomposites reaction yields and estimated mass fraction of GO and QAS in the nanocomposites; Figure S1. Evaluation of antibacterial activity of GO and GO-QAS against E. coli and S. aureus by agar diffusion assay; Figure S2. Evaluation of the antimicrobial activity of GO-QAS against MRSA and MDR-AB; Figure S3. Plate count method results of E. coli and S. aureus after incubation with different concentrations of GO, QAS, and GO-QAS dispersions. Figure S4. Photograph of GO and GO-QAS nanosheets dispersed in different aqueous solutions without sonication. An additional file shows these data [see Additional file 1]. (DOC 10857 kb) [file 41038_2018_115_MOESM1_ESM.doc]

**Additional file 1**

**Synthesis of Graphene Oxide-Quaternary Ammonium Nanocomposite with Synergistic Antibacterial Activity to Promote Infected Wound Healing**

| Samples | GO-NH_2_ (mg) | Poly (AEDMHA) (QAS, mg) | GO-NH_2_: QAS  (mass ratio) | Yield after purification (mg) | Estimated mass fraction of GO in GO-QAS (%, does not contain silane groups) | Estimated mass fraction of QAS in GO-QAS nanocomposites (%) |
| --- | --- | --- | --- | --- | --- | --- |
| GO-QAS-1 | 60 | 120 | 1:2 | 120 | 21.5 | 50.0 |
| GO-QAS-2 | 50 | 200 | 1:4 | 166 | 13.0 | 69.7 |
| GO-QAS-3 | 100 | 800 | 1:8 | 535 | 8.0 | 81.3 |
| GO-QAS-4 | 45 | 720 | 1:16 | 330 | 5.9 | 86.3 |

**Table S1:** The conjugated GO-QAS nanocomposites reaction yields and estimated mass fraction of GO and QAS in the nanocomposites.


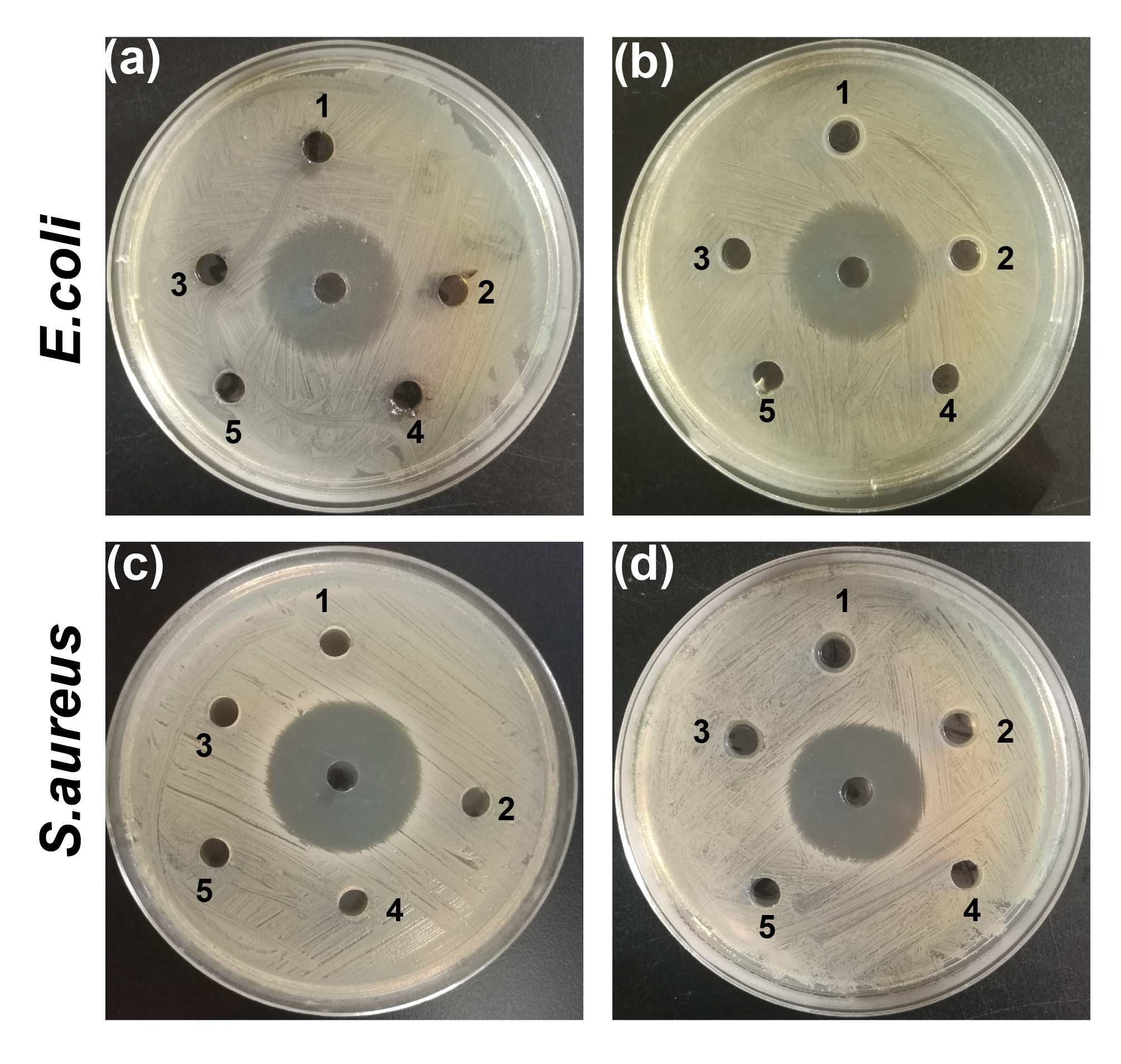


**Figure S1.** Evaluation of antibacterial activity of (a, c) GO and (b, d) GO-QAS against *E. coli* and *S. aureus* by agar diffusion assay. (1: 200
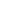
g/mL; 2: 100
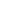
g/mL; 3: 50
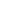
g/mL; 4: 10
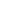
g/mL; 5: 5
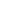
g/mL).


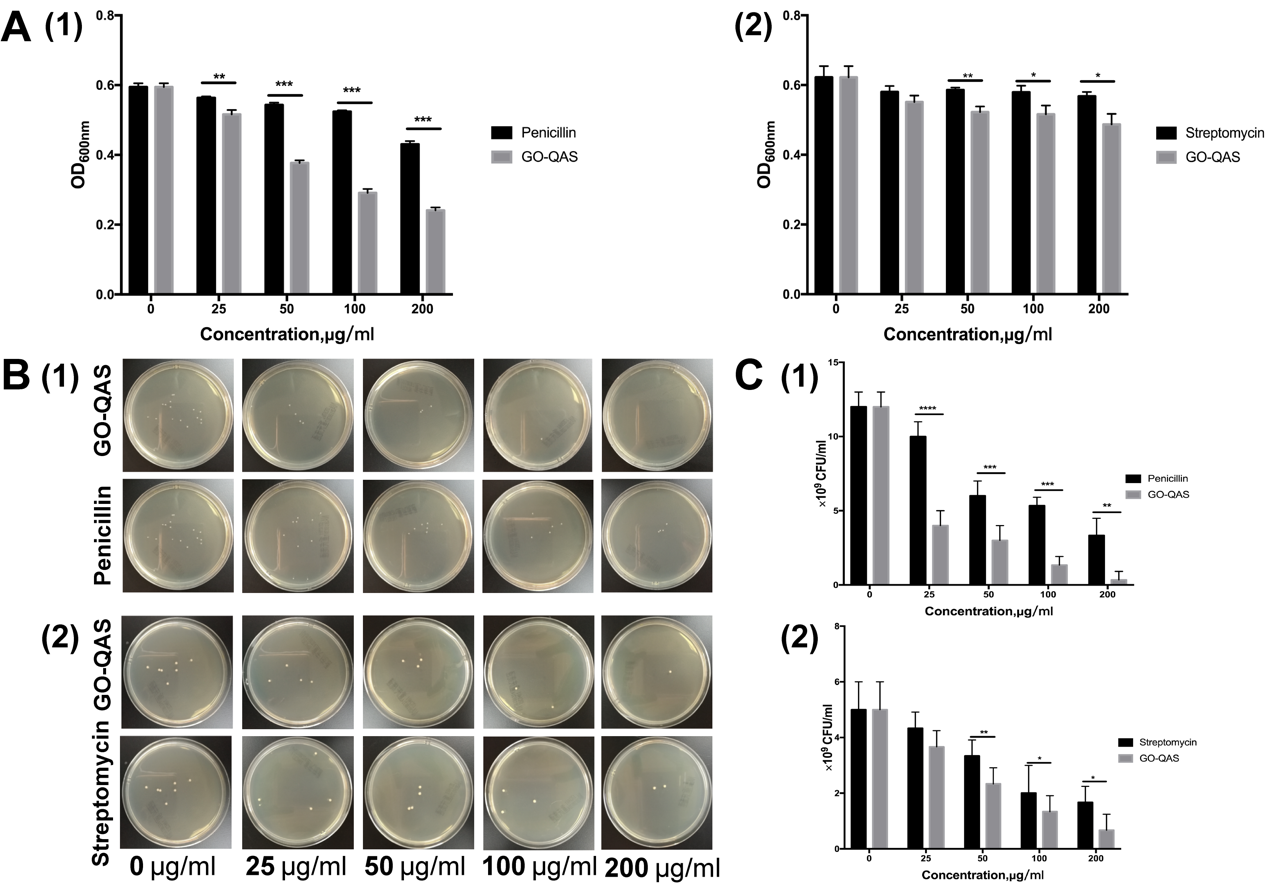


**Figure S2.** Evaluation of the antimicrobial activity of GO-QAS against (1) MRSA and (2) MDR-AB by (A) OD_600nm_ value measurement, and (B) plate count method. (C) Statistical analysis of plate count result. (*represents p < 0.05, **represents p < 0.01, ***represents p < 0.001, ****represents p < 0.0001)


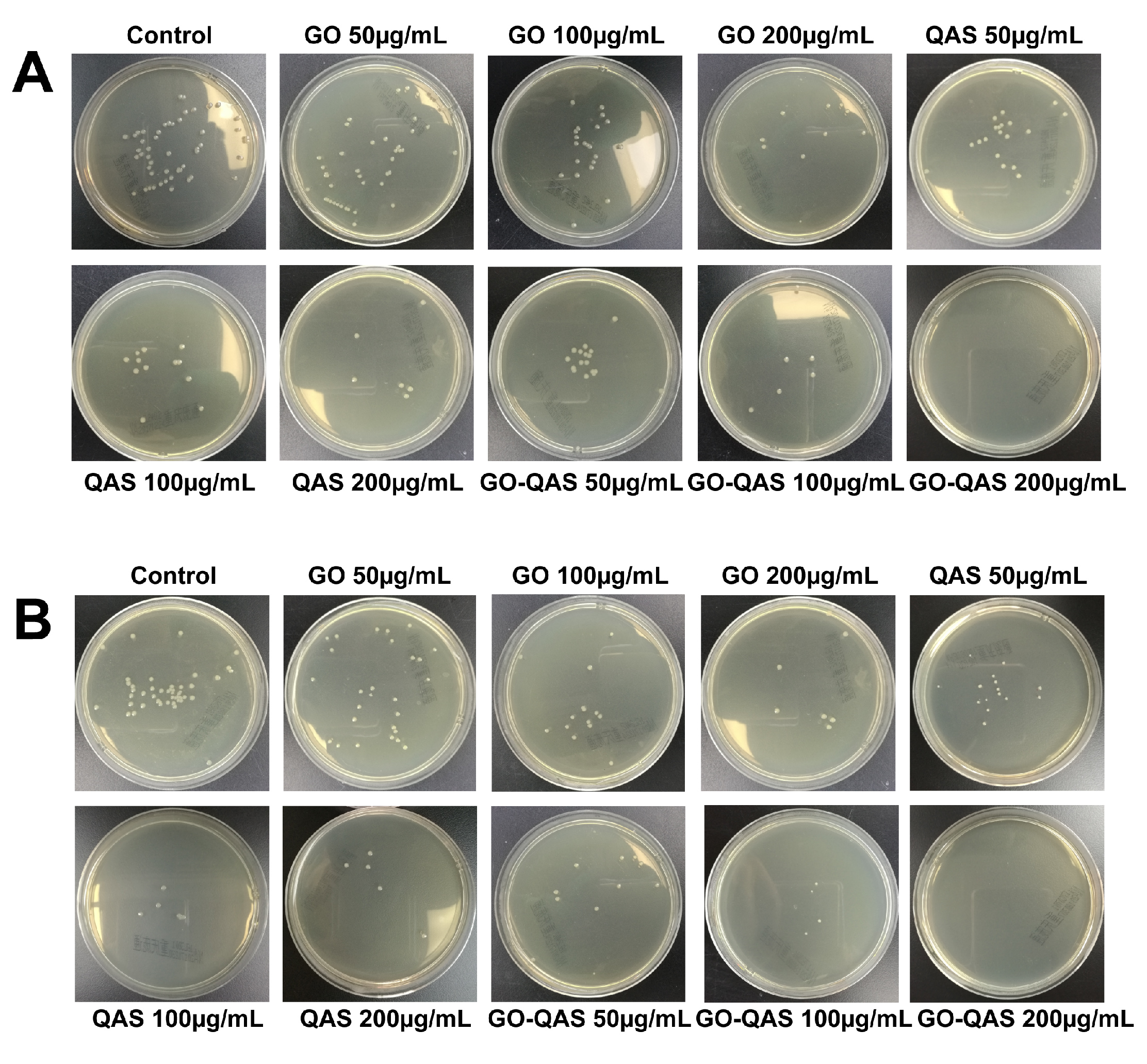


**Figure S3.** Plate count method results of (A) E. coli and (B) S. aureus after incubation with different concentrations of GO, QAS, and GO-QAS dispersions.


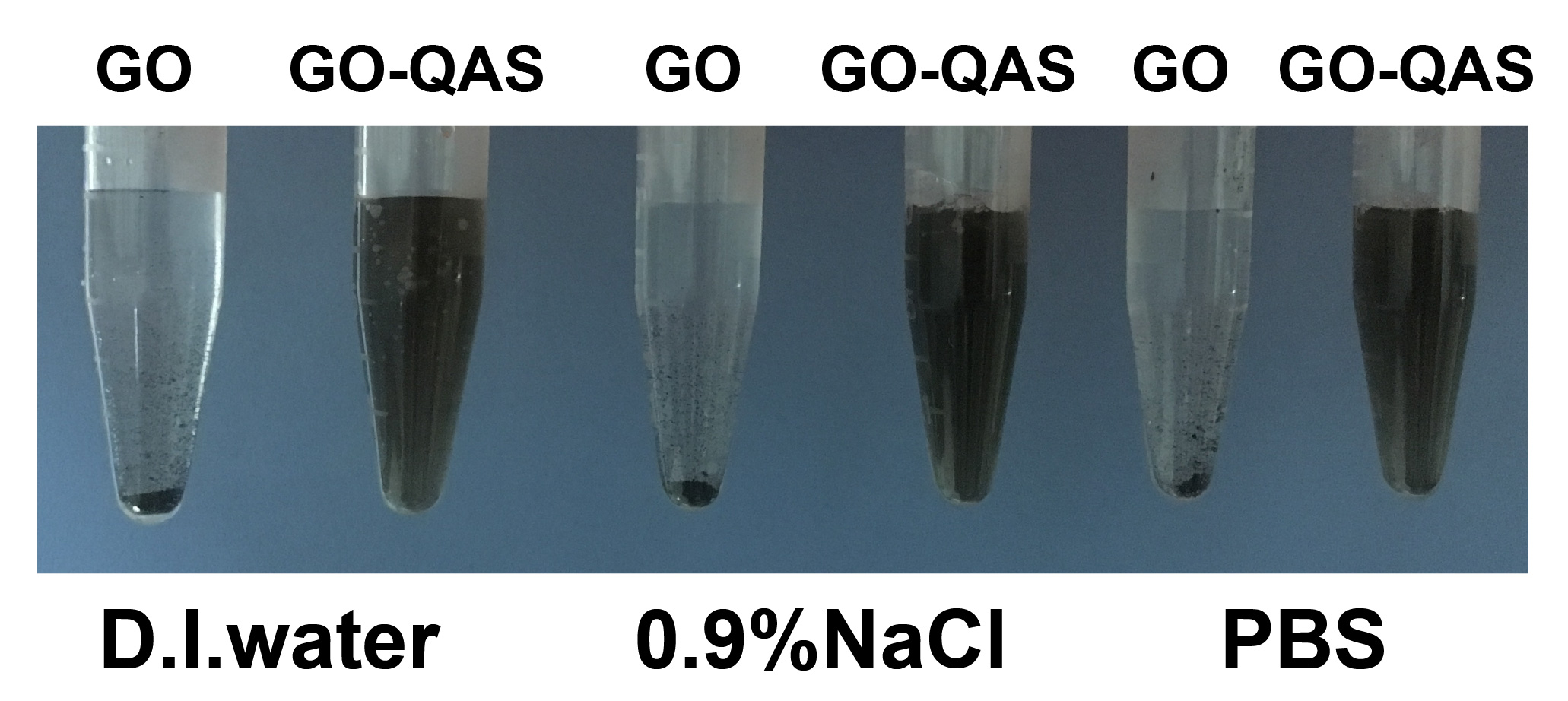


**Figure S4.** Photograph of GO and GO-QAS nanosheets dispersed in different aqueous solutions without sonication.
